# Hopf Bifurcation of an Epidemic Model with Delay

**DOI:** 10.1371/journal.pone.0157367

**Published:** 2016-06-15

**Authors:** Li-Peng Song, Xiao-Qiang Ding, Li-Ping Feng, Qiong Shi

**Affiliations:** Department of Computer Science and Technology, North University of China, Taiyuan, Shan’xi 030051, People’s Republic of China; Shanxi University, CHINA

## Abstract

A spatiotemporal epidemic model with nonlinear incidence rate and Neumann boundary conditions is investigated. On the basis of the analysis of eigenvalues of the eigenpolynomial, we derive the conditions of the existence of Hopf bifurcation in one dimension space. By utilizing the normal form theory and the center manifold theorem of partial functional differential equations (PFDs), the properties of bifurcating periodic solutions are analyzed. Moreover, according to numerical simulations, it is found that the periodic solutions can emerge in delayed epidemic model with spatial diffusion, which is consistent with our theoretical results. The obtained results may provide a new viewpoint for the recurrent outbreak of disease.

## Introduction

Currently, new infectious diseases continuously emerge, and existing diseases recurrently outbreak [1–9]. Ebola virus was firstly discovered in 1976, which began to outbreak in Guinea in February 2014, then spread to West Africa. It caused serious death and social panic. After the outbreak of 2014, Ebola once again emerged in Guinea in March 2016 [10–12]. These diseases have brought a great threat to the public health. In order to provide some suggestions for the prevention and control of the disease, it is necessary to establish rational mathematics model based on infectious mechanism of disease, the route of transmission, and the symptoms of the infected individuals. In particular, the incidence rate describes the number of new infections per unit time, which largely reflects the transmission mechanism of the disease [13–17]. For example, Capasso et al. proposed saturated incidence rate *βSI*/(1 + *kI*) to model the cholera epidemics in Bari in 1973, which reflects the psychological effect or the inhibition effect [18]. By taking appropriate preventive measures, May and Anderson gave nonlinear incidence rate *β*(*SI*/(1 + *αS*)) [19]. Therefore, some reasonable suggestions can be provided for the prevention and effective control of infectious diseases.

It takes an individual a period of time to show the corresponding symptoms based on the infectious mechanism of disease, after an individual is infected disease, such as, dengue, rabies, cholera and so on [20–27]. Therefore, time delay describing the incubation period of disease is a significant quantity. In fact, these potentially asymptomatic individuals (incubation individuals) may promote the wide spread of disease [28, 29]. Thus, it is necessary for us to introduce time delay in the epidemic models.

Because of all the species living in the space environment, and they could diffuse the surrounding area. The individual diffusion in space has an effect on the disease contagion. For example, Zhang et al. indicated that dog movement led to the traveling wave of dog and human rabies and had a large influence on the minimal wave speed [30]. However, previous works on epidemic models did not account for the spatial diffusion factors. McCluskey proved that the endemic equilibrium was globally asymptotically stable whenever it existed for an SIR epidemic model with delay and nonlinear incidence rate [31]. A delayed predator-prey system with disease in the prey was investigated by Han et al., they considered the existence of Hopf bifurcation with time delay in terms of degree 2 [32]. Hence, it is more suitable for us to consider time delay and spatial factor in epidemic model.

This paper is organized as below. In Section II, the eigenpolynomials of spatiotemporal epidemic model with nonlinear incidence rate are given, we further analyze the existence of Hopf bifurcation for two cases. In Section III, by using the normal form theory and the center manifold theorem, some properties of Hopf bifurcation are showed. In Section IV, on the basis of numerical simulations, we show that the epidemics will display recurrent behavior if time delay exceeds a critical point. Finally, some conclusions are obtained.

## Materials and Methods

### Existence of Hopf bifurcation

We consider a SI epidemic model with nonlinear incidence rate *βS*^*p*^*I*^*q*^ with *p* > 0, *q* > 0. This form of nonlinear incidence rate was firstly proposed by Liu et al., and exhibited qualitatively different dynamical behaviors [33, 34]. Therefore, it is helpful to interpret some complex epidemic phenomena. In this paper, let *p* = 1 and *q* = 2. Since the time delay describing incubation period of transmission process widely exists in most epidemiological models [35–37], thus we need to introduce the time delay into the infected population. Furthermore, we consider Neumann boundary conditions. Consequently, the following system with Neumann boundary conditions is given:

$$\begin{array}{c} \left\{\begin{array}{cc} \frac{\partial S(x,t)}{\partial t}= & A-\beta S\left(x,t\right){\left(I(x,t-\tau )\right)}^{2}-dS\left(x,t\right)+d_{1}\nabla^{2}S\left(x,t\right),\\ \frac{\partial I(x,t)}{\partial t}= & \beta S\left(x,t\right){\left(I(x,t-\tau )\right)}^{2}-\left(\mu +d\right)S\left(x,t\right)+d_{2}\nabla^{2}I\left(x,t\right),t\geq 0,x\in \left(0,\pi \right),\\ & \frac{\partial S(x,t)}{\partial x}{|}_{x=0,\pi }=0,\frac{\partial I(x,t)}{\partial x}{|}_{x=0,\pi }=0,\;t\geq 0,\\ & S\left(x,t\right)=\phi_{1}\left(x,t\right)\geq 0,I\left(x,t\right)=\phi_{2}\left(x,t\right)\geq 0,\;\left(x,t\right)\in \left[0,\pi \right]\times \left[-\tau ,0\right], \end{array}\right. \end{array}$$ (1)

where *S*(*x*, *t*) represents the number of the susceptible at location *x* and time *t*, *I*(*x*, *t*) the number of the infectious at location *x* and time *t*, *A* represents the recruitment rate of the susceptible, *d* and *μ* are natural death rate and the disease-related death rate due to the infected, respectively. *d*_1_ and *d*_2_ are diffusion coefficients. *x* represents the one dimensional space, and $\nabla^{2}=\frac{\partial^{2}}{\partial^{2}x}$ denotes the usual Laplacian operator.

Assuming *ϕ* = (*ϕ*_1_, *ϕ*_2_)^*T*^ ∈ ℘ = *C*([−*τ*,0], *X*), *τ* > 0 and *X* is defined as

$$\begin{array}{c} X=\left\{{\left(S(x,t),I(x,t)\right)}^{T}:S\left(x,t\right),I\left(x,t\right)\in W^{2,2}\left(0,\pi \right);\frac{\partial S(x,t)}{\partial x}{|}_{x=0,\pi }=\frac{\partial I(x,t)}{\partial x}{|}_{x=0,\pi }=0\right\} \end{array}$$

with the inner product 〈⋅, ⋅〉.

System (1) without diffusion and delay corresponds to the following system:

$$\begin{array}{c} \left\{\begin{array}{cc} \frac{dS(t)}{dt}= & A-\beta S\left(t\right){\left(I(t)\right)}^{2}-dS\left(t\right),\\ \frac{dI(t)}{dt}= & \beta S\left(t\right){\left(I(t)\right)}^{2}-\left(\mu +d\right)I\left(t\right). \end{array}\right. \end{array}$$ (2)

The system (2) has three equilibria, $E^{0}\left(\frac{A}{d},0\right)$, the saddle

$$\begin{array}{c} E^{1}=\left(\frac{A\beta +\sqrt{A^{2}\beta^{2}-4d^{3}\beta -8d^{2}\beta \mu -4d\beta \mu^{2}}}{2d\beta },\frac{2d(d+\mu )}{A\beta +\sqrt{A^{2}\beta^{2}-4d^{3}\beta -8d^{2}\beta \mu -4d\beta \mu^{2}}}\right), \end{array}$$

and the stable node E*(S*, I*), where

$$\begin{array}{c} S^{*}=\frac{A\beta -\sqrt{A^{2}\beta^{2}-4d^{3}\beta -8d^{2}\beta \mu -4d\beta \mu^{2}}}{2d\beta }, \end{array}$$

$$\begin{array}{c} I^{*}=\frac{2d(d+\mu )}{A\beta -\sqrt{A^{2}\beta^{2}-4d^{3}\beta -8d^{2}\beta \mu -4d\beta \mu^{2}}}. \end{array}$$

Based on the biological meaning, *E*^1^ and E* are satisfied the following conditions [38]:

$$\begin{array}{c} \left(A1\right)\;A^{2}\beta >4d{\left(d+\mu \right)}^{2}. \end{array}$$

Let $\bar{S}=S-S^{*}$, $\bar{I}=I-I^{*}$, then system (1) can be transformed into:

$$\begin{array}{c} \left\{\begin{array}{cc} \frac{\partial \bar{S}}{\partial t}= & A-\beta \left(\bar{S}+S^{*}\right){\left(\bar{I}\left(t-\tau \right)+I^{*}\right)}^{2}-d\left(\bar{S}+S^{*}\right)+d_{1}\nabla^{2}\bar{S},\\ \frac{\partial \bar{I}}{\partial t}= & \beta \left(\bar{S}+S^{*}\right){\left(\bar{I}\left(t-\tau \right)+I^{*}\right)}^{2}-\left(\mu +d\right)\left(\bar{I}+I^{*}\right)+d_{2}\nabla^{2}\bar{I}. \end{array}\right. \end{array}$$ (3)

One can define

$$\begin{array}{l} f^{\left(1\right)}(\bar{S},\bar{I},\bar{I}(t-\tau ))=A-\beta (\bar{S}+S^{*}){\left(\bar{I}(t-\tau )+I^{*}\right)}^{2}-d(\bar{S}+S^{*}),\\ f^{\left(2\right)}(\bar{S},\bar{I},\bar{I}(t-\tau ))=\beta (\bar{S}+S^{*}){\left(\bar{I}(t-\tau )+I^{*}\right)}^{2}-(\mu +d)(\bar{I}+I^{*}), \end{array}$$

and for *i*, *j*, *l* = 0, 1, 2, …, let

$$\begin{array}{c} f_{ijl}^{\left(1\right)}=\frac{\partial^{i+j+l}f^{\left(1\right)}}{\partial {\bar{S}}^{i}\partial {\bar{I}}^{j}{\bar{I}}^{l}\left(t-\tau \right)}\left(0,0,0\right),i+j+l\geq 1,f_{ijl}^{\left(2\right)}=\frac{\partial^{i+j+l}f^{\left(2\right)}}{\partial {\bar{S}}^{i}\partial {\bar{I}}^{j}{\bar{I}}^{l}\left(t-\tau \right)}\left(0,0,0\right),i+j+l\geq 1. \end{array}$$

In the phase space ℘ = *C*([−*τ*,0];*X*), the abstract differential equation of the system (3) is

$$\begin{array}{c} \frac{dU(t)}{dt}=D\Delta U\left(t\right)+L\left(U_{t}\right)+F\left(U_{t}\right), \end{array}$$ (4)

where $U={\left(u_{1},u_{2}\right)}^{T},\bar{S}\left(x,t\right)=u_{1}\left(x,t\right),\bar{I}\left(x,t\right)=u_{2}\left(x,t\right)$, $D=(\begin{array}{cc} d_{1} & 0\\ 0 & d_{2} \end{array}).$ We set *U*_*t*_(*θ*) = *U*(*t*+*θ*),*ϕ* = (*ϕ*_1_, *ϕ*_2_)^*T*^ ∈ ℘, *ϕ*(*θ*) = *U*_*t*_(*θ*), and *θ* ∈ [−*τ*, 0].

Let *L*: ℘ → *X* and *F*: ℘ → *X* are given by

$$\begin{array}{c} L\left(\phi \right)=\left(\begin{array}{c} a_{11}\phi_{1}\left(0\right)+a_{12}\phi_{2}\left(0\right)+a_{13}\phi_{2}\left(-\tau \right)\\ a_{21}\phi_{1}\left(0\right)+a_{22}\phi_{2}\left(0\right)+a_{23}\phi_{2}\left(-\tau \right) \end{array}\right), \end{array}$$

where $a_{11}=f_{100}^{\left(1\right)}\left(0,0,0\right)=-\beta {\left(I^{*}\right)}^{2}-d,a_{12}=f_{010}^{\left(1\right)}\left(0,0,0\right)=0,a_{13}=f_{001}^{\left(1\right)}\left(0,0,0\right)=-2\beta S^{*}I^{*},a_{21}=f_{100}^{\left(2\right)}\left(0,0,0\right)=\beta {\left(I^{*}\right)}^{2},a_{22}=f_{010}^{\left(2\right)}\left(0,0,0\right)=-\left(d+\mu \right),a_{23}=f_{001}^{\left(2\right)}\left(0,0,0\right)=2\beta S^{*}I^{*},$ and

$$F(\phi )=\left(\begin{array}{c} \sum_{i+j+l\geq 2}\frac{1}{i!j!l!}f_{ijl}^{\left(1\right)}(0,0,0)\phi_{1}^{i}(0)\phi_{2}^{j}(0)\phi_{2}^{l}(-\tau )\\ \sum_{i+j+l\geq 2}\frac{1}{i!j!l!}f_{ijl}^{\left(2\right)}(0,0,0)\phi_{1}^{i}(0)\phi_{2}^{j}(0)\phi_{2}^{l}(-\tau ) \end{array}\right),$$

where $f_{101}^{\left(1\right)}\left(0,0,0\right)=-2\beta I^{*}$, $f_{002}^{\left(1\right)}\left(0,0,0\right)=-2\beta S^{*}$, $f_{101}^{\left(2\right)}\left(0,0,0\right)=2\beta I^{*}$, $f_{002}^{\left(2\right)}\left(0,0,0\right)=2\beta S^{*}$, $f_{102}^{\left(1\right)}\left(0,0,0\right)=-2\beta$, $f_{102}^{\left(2\right)}\left(0,0,0\right)=2\beta$.

The linearized part of system (4) is given by

$$\begin{array}{c} \frac{dU(t)}{dt}=D\Delta U\left(t\right)+L\left(U_{t}\right), \end{array}$$ (5)

then we set *U*(*t*) = *ye*^*λt*^, and *y* = (*y*_1_, *y*_2_)^*T*^, hence the characteristic equation is

$$\begin{array}{c} \lambda y-D\frac{\partial^{2}y}{\partial x^{2}}-L\left(e^{\lambda \cdot }y\right)=0, \end{array}$$ (6)

where $y\in \text{dom}(\frac{\partial^{2}}{\partial {\text{x}}^{2}})$ and $y\neq 0,\text{dom}(\frac{\partial^{2}}{\partial {\text{x}}^{2}})\in \text{X}$.

On the basis of the Laplacian operator in the bound domain, $\frac{\partial^{2}}{\partial x^{2}}$ on *X* have eigenvalues −*k*^2^ with the corresponding eigenfunctions $\beta_{k}^{1}=(\begin{array}{c} \text{cos}kx\\ 0 \end{array})$, $\beta_{k}^{2}=(\begin{array}{c} 0\\ \text{cos}kx \end{array})$, *k* ∈ *N*_0_ = {0, 1, 2…}, namely, a basis of the phase space X is ${\left\{\beta_{k}^{1},\beta_{k}^{2}\right\}}_{k=0}^{\infty }$. Thus, for ∀ y ∈ X, y can be expanded as Fourier series in the following form:

$$\begin{array}{c} y=\sum_{k=0}^{\infty }Y_{k}^{T}\left(\begin{array}{c} \beta_{k}^{1}\\ \beta_{k}^{2} \end{array}\right),\;\text{and}\;Y_{k}=\left(\begin{array}{c} \langle y,\beta_{k}^{1}\rangle \\ \langle y,\beta_{k}^{2}\rangle \end{array}\right). \end{array}$$ (7)

Furthermore, through simple computations, we have

$$\begin{array}{c} L\left(\phi^{T}\left(\begin{array}{c} \beta_{k}^{1}\\ \beta_{k}^{2} \end{array}\right)\right)=L{\left(\phi \right)}^{T}\left(\begin{array}{c} \beta_{k}^{1}\\ \beta_{k}^{2} \end{array}\right),\;k\in N_{0}. \end{array}$$ (8)

From the above Eqs (7) and (8), Eq (6) can be written as

$$\begin{array}{c} \sum_{k=0}^{\infty }Y_{k}^{T}\left[\lambda I_{2}+Dk^{2}-\left(\begin{array}{cc} a_{11} & a_{13}e^{-\lambda \tau }\\ a_{21} & a_{22}+a_{23}e^{-\lambda \tau } \end{array}\right)\right]\left(\begin{array}{c} \beta_{k}^{1}\\ \beta_{k}^{2} \end{array}\right)=0, \end{array}$$ (9)

then the eigenpolynomial associated with *λ* of system (1) is given by:

$$\begin{array}{cc} \lambda^{2} & +\left[\left(d_{1}+d_{2}\right)k^{2}-a_{11}-a_{22}-a_{23}e^{-\lambda \tau }\right]\lambda +d_{1}d_{2}k^{4}\\ & -\left(d_{2}a_{11}+d_{1}a_{22}+d_{1}a_{23}e^{-\lambda \tau }\right)k^{2}+a_{11}a_{22}+\left(a_{11}a_{23}-a_{13}a_{21}\right)e^{-\lambda \tau }=0, \end{array}$$ (10)

where *a*_11_ = −*β*(I*)^2^−*d*, *a*_13_ = −2βS* I*, *a*_21_ = *β*(I*)^2^, *a*_22_ = −(*d*+*μ*), *a*_23_ = 2βS* I*, then *a*_11_ *a*_23_−*a*_13_ *a*_21_ < 0 can be derived.

Considering *k* = 0, the eigenpolynomial Eq (10) becomes

$$\begin{array}{c} \lambda^{2}-\left(a_{11}+a_{22}+a_{23}e^{-\lambda \tau }\right)\lambda +a_{11}a_{22}+\left(a_{11}a_{23}-a_{13}a_{21}\right)e^{-\lambda \tau }=0. \end{array}$$ (11)

By replacing *λ* with i*w*(*w* > 0) in Eq (11), then

$$\begin{array}{l} {\left(\text{i}w\right)}^{2}-\left(a_{11}+a_{22}+a_{23}e^{-\text{i}w\tau }\right)\text{i}w+a_{11}a_{22}+\left(a_{11}a_{23}-a_{13}a_{21}\right)e^{-\text{i}w\tau }=0,\\ -w^{2}-\left[a_{11}+a_{22}+a_{23}\left(\text{cos}w\tau -\text{isin}w\tau \right)\right]\text{i}w+a_{11}a_{22}+\left(a_{11}a_{23}-a_{13}a_{21}\right)\left(\text{cos}w\tau -\text{isin}w\tau \right)=0, \end{array}$$

Through separating the real and imaginary parts of above equations, the following equations are obtained:

$$\begin{array}{c} \left\{\begin{array}{cc} -w^{2}+a_{11}a_{22}= & a_{23}w\text{sin}w\tau -\left(a_{11}a_{23}-a_{13}a_{21}\right)\text{cos}w\tau ,\\ -(a_{11}+a_{22})w= & a_{23}w\text{cos}w\tau +\left(a_{11}a_{23}-a_{13}a_{21}\right)\text{sin}w\tau . \end{array}\right. \end{array}$$ (12)

Further, by squaring and adding the two parts of Eq (12), we get

$$\begin{array}{c} w^{4}+Bw^{2}+C=0, \end{array}$$ (13)

where $B=a_{11}^{2}+a_{22}^{2}-a_{23}^{2},C=a_{11}^{2}a_{22}^{2}-{\left(a_{11}a_{23}-a_{13}a_{21}\right)}^{2}>0$. Thus we give

$$\begin{array}{c} w^{2}=\frac{-B+\sqrt{B^{2}-4C}}{2},\;{\hat{w}}^{2}=\frac{-B-\sqrt{B^{2}-4C}}{2}, \end{array}$$

if the formula *B* < 0, and *B*^2^ − 4*C* > 0 then *w*^2^ > 0 and ${\hat{w}}^{2}>0$ can hold simultaneously. Moreover,the corresponding condition is

$$\text{(}A2)\;\beta {\left(I^{*}\right)}^{2}\;+\;d\;-\;\sqrt{3}(d\;+\;\mu )\;<\;0,$$

$$\begin{array}{ll} (A3)\;\beta^{4}{\left(I^{*}\right)}^{8} & \;+\;4d\beta^{3}{\left(I^{*}\right)}^{6}-2\beta^{2}(2d^{2}+10d\mu +5\mu^{2}){\left(I^{*}\right)}^{4}\\ & -\;4d\beta (4d^{2}+10d\mu +5\mu^{2}){\left(I^{*}\right)}^{2}+{\left(4d^{2}+6d\mu +3\mu^{2}\right)}^{2}>0, \end{array}$$

therefore, Eq (11) has two groups of simple imaginary roots ±i*w*_0_, $\pm \text{i}{\hat{w}}_{0}$.

In the following part, we take into account the imaginary roots ±i*w*_0_, the other one is similar.

From Eq (12), we can obtain

$$\begin{array}{c} \text{cos}\left(w_{0}\tau \right)=-\frac{\left(a_{22}a_{23}+a_{13}a_{21}\right)w_{0}^{2}+a_{11}a_{22}\left(a_{11}a_{23}-a_{13}a_{21}\right)}{a_{23}^{2}w_{0}^{2}+{\left(a_{11}a_{23}-a_{13}a_{21}\right)}^{2}}=P\left(w_{0}\right),\\ \text{sin}\left(w_{0}\tau \right)=\frac{w_{0}\left[a_{11}a_{22}a_{23}-a_{23}w_{0}^{2}-\left(a_{11}+a_{22}\right)\left(a_{11}a_{23}-a_{13}a_{21}\right)\right]}{a_{23}^{2}w_{0}^{2}+{\left(a_{11}a_{23}-a_{13}a_{21}\right)}^{2}}=Q\left(w_{0}\right). \end{array}$$

Moreover, some simple derivations show that

$$\tau_{0}^{j}=\{\begin{array}{lll} \frac{1}{w_{0}}\left(\text{arccos}\left(P\left(w_{0}\right)\right)+2j\pi \right), & \text{when} & Q\left(w_{0}\right)\geq 0,\\ \frac{1}{w_{0}}\left(2\pi -\text{arccos}\left(P\left(w_{0}\right)\right)+2j\pi \right), & \text{when} & Q\left(w_{0}\right)\leq 0,j=0,1,2,\ldots_{.} \end{array}$$

*λ*(*τ*) = *α*(*τ*) + i*w*(*τ*) is the root of Eq (11) near $\tau_{0}^{j}$, which satisfies $\alpha (\tau_{0}^{j})=0$ and $w\left(\tau_{0}^{j}\right)=w_{0}$, where *j* = 0, 1, 2, ….

Next, taking the derivative with respect to *τ* on two sides of Eq (11), then we derive

$$\begin{array}{cc} {}[2\lambda & -\left(a_{11}+a_{22}+a_{23}e^{-\lambda \tau }\right)]\frac{d\lambda }{d\tau }+\left[a_{23}\lambda -\left(a_{11}a_{23}-a_{13}a_{21}\right)\right]\tau e^{-\lambda \tau }\frac{d\lambda }{d\tau }\\ & +\left[a_{23}\lambda -\left(a_{11}a_{23}-a_{13}a_{21}\right)\right]\lambda e^{-\lambda \tau }=0. \end{array}$$

By the above expression, one can derive

$$\begin{array}{cc} \text{Re}{\left(\frac{\text{d}\lambda }{\text{d}\tau }\right)}_{\tau =\tau_{0}^{j}}^{-1} & =\text{Re}{\left\{\frac{2\lambda -(a_{11}+a_{22}+a_{23}e^{-\lambda \tau })}{\left[-a_{23}\lambda +\left(a_{11}a_{23}-a_{13}a_{21}\right)\right]\lambda e^{-\lambda \tau }}+\frac{\left[a_{23}\lambda -\left(a_{11}a_{23}-a_{13}a_{21}\right)\right]\tau e^{-\lambda \tau }}{\left[-a_{23}\lambda +\left(a_{11}a_{23}-a_{13}a_{21}\right)\right]\lambda e^{-\lambda \tau }}\right\}}_{\tau =\tau_{0}^{j}}\\ & =\text{Re}{\left\{\frac{2\lambda e^{\lambda \tau }-\left(a_{11}e^{\lambda \tau }+a_{22}e^{\lambda \tau }+a_{23}\right)}{[-a_{23}\lambda +\left(a_{11}a_{23}-a_{13}a_{21}\right)]\lambda }-\frac{\tau }{\lambda }\right\}}_{\tau =\tau_{0}^{j}}\\ & =\{\frac{2w_{0}^{2}\left[\left(a_{11}a_{23}-a_{13}a_{21}\right)\text{cos}w_{0}\tau -a_{23}w_{0}\text{sin}w_{0}\tau \right]-a_{23}^{2}w_{0}^{2}}{\left[a_{23}^{2}w_{0}^{2}+{\left(a_{11}a_{23}-a_{13}a_{21}\right)}^{2}\right]w_{0}^{2}}\\ & \;\;+\frac{-\left(a_{11}+a_{22}\right)w_{0}\left[a_{23}w_{0}\text{cos}w_{0}\tau +\left(a_{11}a_{23}-a_{13}a_{21}\right)\text{sin}w_{0}\tau \right]}{\left[a_{23}^{2}w_{0}^{2}+{\left(a_{11}a_{23}-a_{13}a_{21}\right)}^{2}\right]w_{0}^{2}}{\}}_{\tau =\tau_{0}^{j}}\\ & =\frac{\left[2w_{0}^{2}+a_{11}^{2}+a_{22}^{2}-a_{23}^{2}\right]w_{0}^{2}}{w_{0}^{2}\left[a_{23}^{2}w_{0}^{2}+{\left(a_{11}a_{23}-a_{13}a_{21}\right)}^{2}\right]},\\ & =\frac{2w_{0}^{2}+B}{a_{23}^{2}w_{0}^{2}+{\left(a_{11}a_{23}-a_{13}a_{21}\right)}^{2}}>0,\\ & =\frac{\sqrt{B^{2}-4C}}{a_{23}^{2}w_{0}^{2}+{\left(a_{11}a_{23}-a_{13}a_{21}\right)}^{2}}>0. \end{array}$$

So the transversality condition

$$\begin{array}{c} {\left\{\text{Re}\left(\frac{\text{d}\lambda }{\text{d}\tau }\right)\right\}}_{\tau =\tau_{0}^{j}}>0 \end{array}$$

is deduced.

**Theorem 2.1** If (A1), (A2) and (A3) are all satisfied, system (1) without diffusion experiences a spatially homogeneous Hopf bifurcation at equilibrium E* = (S*, I*) when $\tau =\tau_{0}^{j}$, and period solution will appear.

**Lemma 2.1** (S1) If there is a certain *k*_0_ ∈ *N* = {1, 2, …} such that

$$\begin{array}{c} d_{1}d_{2}k_{0}^{4}-\left(d_{2}a_{11}+d_{1}a_{22}+d_{1}a_{23}\right)k_{0}^{2}+a_{11}a_{22}+\left(a_{11}a_{23}-a_{13}a_{21}\right)<0, \end{array}$$ (14)

then Eq (10) has a pair purely imaginary roots ±i*w*_*k*_0__, and

$$\begin{array}{c} w_{k_{0}}=\frac{\sqrt{2}}{2}\sqrt{-B_{k_{0}}+\sqrt{B_{k_{0}}^{2}-4C_{k_{0}}}}. \end{array}$$ (15)

**Proof:** If we assume *k* = *k*_0_ ∈ *N*, and *λ* = i*w*(*w* > 0) be a root of Eq (10). By inserting i*w*(*w* > 0) into Eq (10) and using the same method as before, then Eq (10) can be translated into:

$$\begin{array}{c} w^{4}+Bw^{2}+C=0, \end{array}$$ (16)

where $B=\left(d_{1}^{2}+d_{2}^{2}\right)k^{4}-2d_{1}a_{11}k^{2}-2d_{2}a_{22}k^{2}+a_{11}^{2}+a_{22}^{2}-a_{23}^{2}$, *C* = [*d*_1_ *d*_2_ *k*^4^−*d*_2_ *a*_11_ *k*^2^−*d*_1_ *a*_23_ *k*^2^+*a*_11_ *a*_23_]^2^−[*d*_1_ *a*_22_ *k*^2^−(*a*_11_ *a*_22_−*a*_12_ *a*_21_)]^2^, for ∀*k* ∈ *N*. Besides, we set *C* = *C*_1_ × *C*_2_, where

$$\begin{array}{cc} & C_{1}=d_{1}d_{2}k^{4}-\left(d_{2}a_{11}+d_{1}a_{22}-d_{1}a_{23}\right)k^{2}+a_{11}a_{22}-\left(a_{11}a_{23}-a_{13}a_{21}\right),\\ & C_{2}=d_{1}d_{2}k^{4}-\left(d_{2}a_{11}+d_{1}a_{22}+d_{1}a_{23}\right)k^{2}+a_{11}a_{22}+\left(a_{11}a_{23}-a_{13}a_{21}\right). \end{array}$$

Further, we can deduce

$$\begin{array}{c} w^{2}=\frac{-B\pm \sqrt{B^{2}-4C}}{2}. \end{array}$$ (17)

It is clear that *C*_1_(*k*) > 0 for ∀*k* ∈ *N*, according to assumption (S1), *C*(*k*_0_) < 0 is given, then we get $w_{k_{0}}^{2}>0$,

$$\begin{array}{c} w_{k_{0}}=\frac{\sqrt{2}}{2}\sqrt{-B_{k_{0}}+\sqrt{B_{k_{0}}^{2}-4C_{k_{0}}}}. \end{array}$$

Lemma 2.1 shows that the critical value of bifurcation parameter *τ* can be found. Similar to the method for the case of *k* = 0,

$$\begin{array}{cc} \text{cos}(w_{k_{0}}\tau )= & \frac{d_{1}^{2}d_{2}a_{23}k_{0}^{6}+\left[-d_{1}d_{2}\left(2a_{11}a_{23}-a_{13}a_{21}\right)-d_{1}^{2}a_{22}a_{23}\right]k_{0}^{4}}{a_{23}^{2}w_{k_{0}}^{2}+{\left[d_{1}a_{23}k_{0}^{2}-\left(a_{11}a_{23}-a_{13}a_{21}\right)\right]}^{2}}\\ & +\frac{d_{2}\left[a_{11}\left(a_{11}a_{23}-a_{13}a_{21}\right)+a_{23}w_{k_{0}}^{2}\right]k_{0}^{2}+d_{1}a_{22}\left(2a_{11}a_{23}-a_{13}a_{21}\right)k_{0}^{2}}{a_{23}^{2}w_{k_{0}}^{2}+{\left[d_{1}a_{23}k_{0}^{2}-\left(a_{11}a_{23}-a_{13}a_{21}\right)\right]}^{2}}\\ & +\frac{-a_{11}a_{22}\left(a_{11}a_{23}-a_{13}a_{21}\right)-a_{13}a_{21}w_{k_{0}}^{2}-a_{22}a_{23}w_{k_{0}}^{2}}{a_{23}^{2}w_{k_{0}}^{2}+{\left[d_{1}a_{23}k_{0}^{2}-\left(a_{11}a_{23}-a_{13}a_{21}\right)\right]}^{2}}=X\left(w_{k_{0}}\right),\\ \text{sin}(w_{k_{0}}\tau )= & \frac{-d_{1}^{2}a_{23}w_{k_{0}}k_{0}^{4}+\left[d_{1}\left(2a_{11}a_{23}-a_{13}a_{21}\right)-d_{2}a_{13}a_{21}\right]w_{k_{0}}k_{0}^{2}}{a_{23}^{2}w_{k_{0}}^{2}+{\left[d_{1}a_{23}k_{0}^{2}-\left(a_{11}a_{23}-a_{13}a_{21}\right)\right]}^{2}}\\ & +\frac{-\left(a_{11}a_{23}-a_{13}a_{21}\right)a_{11}w_{k_{0}}+a_{13}a_{21}a_{22}w_{k_{0}}-a_{23}w_{k_{0}}^{3}}{a_{23}^{2}w_{k_{0}}^{2}+{\left[d_{1}a_{23}k_{0}^{2}-\left(a_{11}a_{23}-a_{13}a_{21}\right)\right]}^{2}}=Y\left(w_{k_{0}}\right), \end{array}$$

then we get

$$\begin{array}{c} \tau_{k_{0}}^{j}=\left\{\begin{array}{cc} & \frac{1}{w_{k_{0}}}\left(\text{arccos}\left(X\left(w_{k_{0}}\right)\right)+2j\pi \right),\;\;\;\;\text{when}\;Y\left(w_{k_{0}}\right)\geq 0,\\ & \frac{1}{w_{k_{0}}}\left(2\pi -\text{arccos}\left(X\left(w_{k_{0}}\right)\right)+2j\pi \right),\;\;\text{when}\;Y\left(w_{k_{0}}\right)\leq 0,j=0,1,2,..._{.} \end{array}\right. \end{array}$$

Let *λ*(*τ*) = *α*(*τ*) + i*w*(*τ*) be the root of (10) near $\tau_{k_{0}}^{j}$ which satisfies $\alpha (\tau_{k_{0}}^{j})=0$ and $w\left(\tau_{k_{0}}^{j}\right)=w_{k_{0}}$, where *j* = 0, 1, 2, ….

**Lemma 2.2** If condition (S1) is established, then the transversality condition

$$\begin{array}{c} {\left\{\text{Re}\left(\frac{d\lambda }{d\tau }\right)\right\}}_{\tau =\tau_{k_{0}}^{j}}>0 \end{array}$$

is derived.

The proof can be found in S1 File.

**Theorem 2.2** In the presence of space, if the conditions (A1) and (S1) are satisfied, then system (1) undergoes a Hopf bifurcation at E* = (S*, I*) when $\tau =\tau_{k_{0}}^{j}$, and period solution will emerge.

## Results

### The properties of Hopf bifurcating period solutions

The above section gives the conditions of the existence of Hopf bifurcation for two cases. In this section, we investigate properties of these bifurcating periodic solutions from the positive constant steady state E*(S*, I*) of system (1) by employing the normal form theory and the center manifold theorem of partial functional differential equations (PFDEs) [39–42], these properties include the direction, stability and period. It’s simple for mathematical calculation to mark $\tau_{c}=\tau_{k}^{j}\left(k=0,k_{0};j=0,1,2,...\right)$.

Let $\hat{S}\left(t,x\right)=\bar{S}\left(\tau t,x\right)$, $\hat{I}\left(t,x\right)=\bar{I}\left(\tau t,x\right)$, then system (3) can be expressed as

$$\begin{array}{c} \left\{\begin{array}{cc} \frac{\partial \hat{S}\left(x,t\right)}{\partial t}= & \tau d_{1}\frac{\partial^{2}\hat{S}\left(x,t\right)}{\partial x^{2}}+\tau \left[a_{11}\hat{S}\left(x,t\right)+a_{13}\hat{I}\left(x,t-1\right)\right]\\ & \;\;\;\;\;\;+\tau \sum_{i+j+l\geq 2}\frac{1}{i!j!l!}f_{ijl}^{\left(1\right)}\left(0,0,0\right){\hat{S}}^{i}\left(x,t\right){\hat{I}}^{j}\left(x,t\right){\hat{I}}^{j}\left(x,t-1\right),\\ \frac{\partial \hat{I}\left(x,t\right)}{\partial t}= & \tau d_{2}\frac{\partial^{2}\hat{I}\left(x,t\right)}{\partial x^{2}}+\tau \left[a_{21}\hat{S}\left(x,t\right)+a_{22}\hat{I}\left(x,t\right)+a_{23}\hat{I}\left(x,t-1\right)\right]\\ & \;\;\;\;\;\;+\tau \sum_{i+j+l\geq 2}\frac{1}{i!j!l!}f_{ijl}^{\left(2\right)}\left(0,0,0\right){\hat{S}}^{i}\left(x,t\right){\hat{I}}^{j}\left(x,t\right){\hat{I}}^{j}\left(x,t-1\right). \end{array}\right. \end{array}$$ (18)

In the space ℘ = *C*([−1, 0], *X*), let *τ* = *τ*_*c*_ + *α* (*α* ∈ *R*), $U={\left(u_{1},u_{2}\right)}^{T},\hat{S}\left(x,t\right)=u_{1}\left(x,t\right),\hat{I}\left(x,t\right)=u_{2}\left(x,t\right)$, then system (18) can be rewritten as:

$$\begin{array}{c} \frac{dU(t)}{dt}=\tau_{c}D\Delta U\left(t\right)+L\left(\tau_{c}\right)U_{t}+F\left(U_{t},\alpha \right). \end{array}$$ (19)

Let *ϕ* = (*ϕ*_1_, *ϕ*_2_)^*T*^ ∈ ℘, *U*_*t*_(*θ*) = *U*(*t*+*θ*), and *ϕ*(*θ*) = *U*_*t*_(*θ*) for *θ* ∈ [−1, 0]. Defining *L*(*b*)(⋅): *R* × ℘ → *X* (*b* is *τ*_*c*_ or *α*) and *F*: ℘ × *R* → *X* as

$$\begin{array}{c} L\left(b\right)\left(\phi \right)=b\left(\begin{array}{c} a_{11}\phi_{1}\left(0\right)+a_{13}\phi_{2}\left(-1\right)\\ a_{21}\phi_{1}\left(0\right)+a_{22}\phi_{2}\left(0\right)+a_{23}\phi_{2}\left(-1\right) \end{array}\right), \end{array}$$

and

$$\begin{array}{c} F(\phi ,\alpha )=aD\Delta \phi (0)+L(\alpha )(\phi )+f(\phi ,\alpha ), \end{array}$$

where

$$f(\phi ,\alpha )=(\tau_{c}+\alpha )\left(\begin{array}{c} \sum_{i+j+l\geq 2}\frac{1}{i!j!l!}f_{ijl}^{\left(1\right)}(0,0,0)\phi_{1}^{i}(0)\phi_{2}^{j}(0)\phi_{2}^{l}(-1)\\ \sum_{i+j+l\geq 2}\frac{1}{i!j!l!}f_{ijl}^{\left(2\right)}(0,0,0)\phi_{1}^{i}(0)\phi_{2}^{j}(0)\phi_{2}^{l}(-1) \end{array}\right).$$

Next, the linear part of the system (19) is given by

$$\begin{array}{c} \dot{U}\left(t\right)=\tau_{c}D\Delta U\left(t\right)+L\left(\tau_{c}\right)U_{t}. \end{array}$$ (20)

From the conclusions of section II, an equilibrium of the system (20) is the origin, the corresponding characteristic equation of the system (20) at origin has two pairs of purely imaginary eigenvalues ±i*w*_0_ *τ*_*c*_, $\pm \text{i}{\hat{w}}_{0}\tau_{c}$ for *k* = 0, and only a pair of purely imaginary eigenvalues ±i*w*_*k*_ *τ*_*c*_ for *k* ∈ *N*. We account for purely imaginary eigenvalues ±i*w*_0_ *τ*_*c*_ for the case *k* = 0, and set Λ_0_ = {i*w*_*k*_ *τ*_*c*_, −i*w*_*k*_ *τ*_*c*_}, (*k* = 0, *k*_0_).

Considering the ordinary functional differential equation:

$$\begin{array}{c} \dot{X}\left(t\right)=-\tau_{c}Dk^{2}X\left(t\right)+L\left(\tau_{c}\right)\left(X_{t}\right). \end{array}$$ (21)

For *ϕ* ∈ *C*([−1, 0], *X*), according to the Riesz representation theorem, there is a 2 × 2 matrix function *η*(*θ*, *τ*_*c*_)(−1 ≤ *θ* ≤ 0), then we have [40]

$$\begin{array}{c} -\tau_{c}Dk^{2}\phi \left(0\right)+L\left(\tau_{c}\right)\left(\phi \right)=\int_{-1}^{0}d\left[\eta \left(\theta ,\tau_{c}\right)\right]\phi \left(\theta \right), \end{array}$$ (22)

where

$$\begin{array}{c} \eta \left(\theta ,\tau_{c}\right)=\left\{\begin{array}{cc} & \tau_{c}\left(\begin{array}{cc} -d_{1}k^{2}+a_{11} & 0\\ a_{21} & -d_{2}k^{2}+a_{22} \end{array}\right),\;\theta =0,\\ & \;\;\;0,\;\;\;\;\;\;\;\;\;\;\;\;\theta \in (-1,0),\\ & \tau_{c}\left(\begin{array}{cc} 0 & -a_{13}\\ 0 & -a_{23} \end{array}\right),\;\;\;\;\;\;\;\theta =-1. \end{array}\right. \end{array}$$ (23)

For *ϕ* ∈ *C*([−1, 0], *X*), defining semigroup induced by the solution of the linear eq (20), and the infinitesimal generator *A*(*τ*_*c*_) of the semigroup is

$$\begin{array}{c} A\left(\tau_{c}\right)\phi \left(\theta \right)=\left\{\begin{array}{cc} & \frac{d\phi (\theta )}{d\theta },\;\;\;\;\;\;\theta \in \left[-1,0\right),\\ & \int_{-1}^{0}d_{\theta }\eta \left(\theta ,\tau_{c}\right)\phi \left(\theta \right),\;\theta =0. \end{array}\right. \end{array}$$ (24)

For *ψ* ∈ *C*([0, 1], *X*), the formal adjoint operators of *A*(*τ*_*c*_) is A*(*τ*_*c*_) which denotes [43]

$$\begin{array}{c} A^{*}\left(\tau_{c}\right)\psi \left(s\right)=\left\{\begin{array}{cc} & -\frac{d\psi (s)}{ds},\;\;\;\;\;\;\;s\in \left(0,1\right],\\ & \int_{-1}^{0}d_{s}\eta \left(s,\tau_{c}\right)\psi \left(-s\right),\;\;s=0. \end{array}\right. \end{array}$$ (25)

Here, the bilinear pairing form associated *A*(*τ*_*c*_) with A*(*τ*_*c*_) is

$$\begin{array}{cc} \left(\psi ,\phi \right) & =\psi \left(0\right)\phi \left(0\right)-\int_{-1}^{0}\int_{\xi =0}^{\theta }\psi \left(\xi -\theta \right)d\eta \left(\theta ,\tau_{c}\right)\phi \left(\xi \right)d\xi \\ & =\psi \left(0\right)\phi \left(0\right)-\tau_{c}\int_{-1}^{0}\psi \left(\xi +1\right)\left(\begin{array}{cc} 0 & -a_{13}\\ 0 & -a_{23} \end{array}\right)\phi \left(\xi \right)d\xi . \end{array}$$ (26)

On the basis of the discussion of section II, *A*(*τ*_*c*_) has a pair purely imaginary eigenvalues ±i*w*_*k*_ *τ*_*c*_, which are also eigenvalues of A*(*τ*_*c*_). Furthermore, the generalized eigenspaces of *A*(*τ*_*c*_) and A*(*τ*_*c*_) associated with Λ_0_ are the center subspaces *P* and P*, respectively. P* is the adjoint space of *P* and dim*P* = dimP* = 2 [42].

By some computations, the following Lemma is directly given:

**Lemma 2.3** A basis of *P* with Λ_0_ is

$$\begin{array}{c} q_{1}\left(\theta \right)=e^{\text{i}w_{k}\tau_{c}\theta }{\left(1,\xi \right)}^{T},\;q_{2}\left(\theta \right)=\bar{q_{1}\left(\theta \right)},\;-1\leq \theta \leq 0, \end{array}$$

and a basis of P* with Λ_0_ is

$$\begin{array}{c} q_{1}^{*}\left(s\right)=\left(1,\eta \right)e^{-\text{i}w_{k}\tau_{c}s},\;q_{2}^{*}\left(s\right)=\bar{q_{1}^{*}\left(s\right)},\;0\leq s\leq 1, \end{array}$$

where

$$\begin{array}{c} \xi =\frac{\left(\text{i}w_{k}+d_{1}k^{2}-a_{11}\right)}{a_{13}e^{-\text{i}w_{k}\tau_{c}}},\;\eta =\frac{\left(\text{i}w_{k}+d_{1}k^{2}-a_{11}\right)}{a_{21}}. \end{array}$$ (27)

Φ = (Φ_1_, Φ_2_) and $\Phi^{*}={\left(\Phi_{1}^{*},\Phi_{2}^{*}\right)}^{T}$ are obtained by separating the real and imaginary parts of *q*_1_(*θ*) and $q_{1}^{*}\left(s\right)$, respectively. Obviously, Φ is the basis of *P*, Φ* is the basis of P*, and

$$\begin{array}{ll} \Phi_{1}(\theta )= & \left(\begin{array}{c} \text{Re}\{e^{\text{i}w_{k}\tau_{c}\theta }\}\\ \text{Re}\{\xi e^{\text{i}w_{k}\tau_{c}\theta }\} \end{array}\right)=\left(\begin{array}{c} \text{cos}w_{k}\tau_{c}\theta \\ \frac{\left(d_{1}k^{2}-a_{11})\text{cos}w_{k}\tau_{c}(\theta +1)-w_{k}\text{sin}w_{k}\tau_{c}(\theta +1\right)}{a_{13}} \end{array}\right),\\ \Phi_{2}(\theta )= & \left(\begin{array}{c} \text{Im}\{e^{\text{i}w_{k}\tau_{c}\theta }\}\\ \text{Im}\{\xi e^{\text{i}w_{k}\tau_{c}\theta }\} \end{array}\right)=\left(\begin{array}{c} \text{sin}w_{k}\tau_{c}\theta \\ \frac{\left(d_{1}k^{2}-a_{11})\text{sin}w_{k}\tau_{c}(\theta +1)+w_{k}\text{cos}w_{k}\tau_{c}(\theta +1\right)}{a_{13}} \end{array}\right).\\ \Phi_{1}^{*}(s)= & {\left(\begin{array}{c} \text{Re}\{e^{-\text{i}w_{k}\tau_{c}s}\}\\ \text{Re}\{\eta e^{-\text{i}w_{k}\tau_{c}s}\} \end{array}\right)}^{T}={\left(\begin{array}{c} \text{cos}w_{k}\tau_{c}s\\ \frac{w_{k}\text{sin}w_{k}\tau_{c}s+(d_{1}k^{2}-a_{11})\text{cos}w_{k}\tau_{c}s}{a_{21}} \end{array}\right)}^{T},\\ \Phi_{2}^{*}(s)== & {\left(\begin{array}{c} \text{Im}\{e^{-\text{i}w_{k}\tau_{c}s}\}\\ \text{Im}\{\eta e^{-\text{i}w_{k}\tau_{c}s}\} \end{array}\right)}^{T}={\left(\begin{array}{c} \text{sin}w_{k}\tau_{c}s\\ \frac{w_{k}\text{cos}w_{k}\tau_{c}s-(d_{1}k^{2}-a_{11})\text{sin}w_{k}\tau_{c}s}{a_{21}} \end{array}\right)}^{T}. \end{array}$$

According to the bilinear pairing form Eq (26), we can compute:

$$\begin{array}{cc} (\Phi_{1}^{*},\Phi_{1})=1 & +\frac{{\left(d_{1}k^{2}-a_{11}\right)}^{2}\text{cos}w_{k}\tau_{c}-w_{k}\left(d_{1}k^{2}-a_{11}\right)\text{sin}w_{k}\tau_{c}}{a_{13}a_{21}}\\ & +\frac{1}{2}\tau_{c}\left[a_{13}a_{21}\left(d_{1}k^{2}-a_{11}\right)+a_{23}{\left(d_{1}k^{2}-a_{11}\right)}^{2}\right]\left(1+\frac{\text{sin}2w_{k}\tau_{c}}{2w_{k}\tau_{c}}\right)\\ & -\frac{1}{2}a_{13}a_{21}{\left(\text{sin}w_{k}\tau_{c}\right)}^{2}-\frac{1}{2}\tau_{c}a_{23}w_{k}^{2}\left(1-\frac{\text{sin}2w_{k}\tau_{c}}{2w_{k}\tau_{c}}\right), \end{array}$$

$$\begin{array}{cc} (\Phi_{1}^{*},\Phi_{2})= & \frac{{\left(d_{1}k^{2}-a_{11}\right)}^{2}\text{sin}w_{k}\tau_{c}+w_{k}\left(d_{1}k^{2}-a_{11}\right)\text{cos}w_{k}\tau_{c}}{a_{13}a_{21}}\\ & +\tau_{c}\frac{a_{23}w_{k}\left(d_{1}k^{2}-a_{11}\right)}{a_{13}a_{21}}+\frac{1}{2}\tau_{c}w_{k}\left(1+\frac{\text{sin}2w_{k}\tau_{c}}{2w_{k}\tau_{c}}\right)\\ & +\tau_{c}\frac{a_{13}a_{21}\left(d_{1}k^{2}-a_{11}\right)+a_{23}\left[w_{k}^{2}+{\left(d_{1}k^{2}-a_{11}\right)}^{2}\right]}{a_{13}a_{21}}\frac{{\left(\text{sin}w_{k}\tau_{c}\right)}^{2}}{2w_{k}\tau_{c}}, \end{array}$$

$$\begin{array}{cc} (\Phi_{2}^{*},\Phi_{1})= & \frac{w_{k}\left(d_{1}k^{2}-a_{11}\right)\text{cos}w_{k}\tau_{c}-w_{k}^{2}\text{sin}w_{k}\tau_{c}}{a_{13}a_{21}}\\ & +\tau_{c}\frac{a_{23}w_{k}\left(d_{1}k^{2}-a_{11}\right)}{a_{13}a_{21}}-\frac{1}{2}\tau_{c}w_{k}\left(1-\frac{\text{sin}2w_{k}\tau_{c}}{2w_{k}\tau_{c}}\right)\\ & +\tau_{c}\frac{a_{13}a_{21}\left(d_{1}k^{2}-a_{11}\right)-a_{23}\left[w_{k}^{2}+{\left(d_{1}k^{2}-a_{11}\right)}^{2}\right]}{a_{13}a_{21}}\frac{{\left(\text{sin}w_{k}\tau_{c}\right)}^{2}}{2w_{k}\tau_{c}}, \end{array}$$

$$\begin{array}{cc} (\Phi_{2}^{*},\Phi_{2})= & \frac{w_{k}\left(d_{1}k^{2}-a_{11}\right)\text{sin}w_{k}\tau_{c}+{\left(w_{k}\right)}^{2}\text{cos}w_{k}\tau_{c}}{a_{13}a_{21}}\\ & +\frac{1}{2}\tau_{c}\left\{\frac{1}{\tau_{c}}{\left(\text{sin}w_{k}\tau_{c}\right)}^{2}+\frac{a_{23}w_{k}^{2}}{a_{13}a_{21}}\left(1+\frac{\text{sin}2w_{k}\tau_{c}}{2w_{k}\tau_{c}}\right)\right\}\\ & +\frac{1}{2}\tau_{c}\frac{a_{13}a_{21}\left(d_{1}k^{2}-a_{11}\right)-a_{23}{\left(d_{1}k^{2}-a_{11}\right)}^{2}}{a_{13}a_{21}}\left(1-\frac{\text{sin}2w_{k}\tau_{c}}{2w_{k}\tau_{c}}\right). \end{array}$$

Next, we construct a new basis Ψ for P*, where Ψ = (Ψ_1_, Ψ_2_)^*T*^ = (Φ*, Φ)^−1^ Φ* and $\left(\Phi^{*},\Phi \right)=\left(\Phi_{ı}^{*},\Phi_{\ell }\right),\left(ı,\ell =1,2\right)$. (Ψ, Φ) = *I*_2_ needs to be satisfied. In addition, $f_{k}=\left(\beta_{k}^{1},\beta_{k}^{2}\right)$, where $\beta_{k}^{1}=(\begin{array}{c} \text{cos}kx\\ 0 \end{array})$, $\beta_{k}^{2}=(\begin{array}{c} 0\\ \text{cos}kx \end{array})$. For *c* = (*c*_1_, *c*_2_) ∈ *C*([−1, 0], *X*), we define $c\cdot f_{k}=c_{1}\beta_{k}^{1}+c_{2}\beta_{k}^{2}$.

On the basis of the theory of decomposition of the phase space, we have ℘ = *P*_*CN*_℘ + *P*_*s*_℘, where *P*_*CN*_℘ is the center subspace of linear Eq (20),

$$\begin{array}{c} P_{CN}℘\left(\phi \right)=\Phi \left(\Psi ,\langle \phi ,f_{k}\rangle \right)\cdot f_{k},\;\phi \in ℘, \end{array}$$ (28)

and *P*_*s*_℘ is the complement subspace of *P*_*CN*_℘.

Since the infinitesimal generator *A*(*τ*_*c*_) is induced by the solution of Eq (20), then Eq (18) can be translated into:

$$\begin{array}{c} {\dot{U}}_{t}=A\left(\tau_{c}\right)U_{t}+X_{0}F\left(U_{t},\alpha \right), \end{array}$$ (29)

where $X_{0}\left(\theta \right)=\{\begin{array}{cc} & 0,\;\;\;\theta \in [-1,0),\\ & I,\;\;\;\theta =0. \end{array}$

According to the phase space decomposition ℘ = *P*_*CN*_℘ + *P*_*s*_℘ and Eq (28), the solution of Eq (19) is written as

$$\begin{array}{c} U_{t}=\Phi \left(\begin{array}{c} x_{1}\left(t\right)\\ x_{2}\left(t\right) \end{array}\right)\cdot f_{k}+h\left(x_{1},x_{2},\alpha \right), \end{array}$$ (30)

where $(\begin{array}{c} x_{1}\left(t\right)\\ x_{2}\left(t\right) \end{array})=\left(\Psi ,\langle U_{t},f_{k}\rangle \right)$, and *h*(*x*_1_, *x*_2_, *α*) ∈ *P*_*s*_℘, *h*(0,0,0) = 0, *Dh*(0,0,0) = 0. Moreover, the solution of Eq (19) on center manifold is

$$\begin{array}{c} U_{t}=\Phi \left(\begin{array}{c} x_{1}\left(t\right)\\ x_{2}\left(t\right) \end{array}\right)\cdot f_{k}+h\left(x_{1},x_{2},0\right). \end{array}$$ (31)

Let *z* = *x*_1_ − *ix*_2_, Ψ(0) = (Ψ_1_(0), Ψ_2_(0))^*T*^, and *q*_1_ = Φ_1_ + iΦ_2_, thus

$$\begin{array}{c} \Phi \left(\begin{array}{c} x_{1}\left(t\right)\\ x_{2}\left(t\right) \end{array}\right)\cdot f_{k}=\left(\Phi_{1},\Phi_{2}\right)\left(\begin{array}{c} \frac{z+\bar{z}}{2}\\ \frac{\text{i}(z-\bar{z})}{2} \end{array}\right)\cdot f_{k}=\frac{1}{2}\left(q_{1}z+\bar{q_{1}}\bar{z}\right)\cdot f_{k}. \end{array}$$ (32)

By using the previous variable substitution, Eq (31) can be transformed into:

$$\begin{array}{c} U_{t}=\frac{1}{2}\left(q_{1}z+\bar{q_{1}}\bar{z}\right)\cdot f_{k}+W\left(z,\bar{z}\right), \end{array}$$ (33)

where $W\left(z,\bar{z}\right)=h(\frac{z+\bar{z}}{2},\frac{\text{i}(z-\bar{z})}{2},0)$, and setting

$$\begin{array}{c} W\left(z,\bar{z}\right)=W_{20}\frac{z^{2}}{2}+W_{11}z\bar{z}+W_{02}\frac{{\bar{z}}^{2}}{2}+..._{.} \end{array}$$ (34)

According to the conclusions of Ref. [42], z satisfies

$$\begin{array}{c} \dot{z}=\text{i}w_{k}\tau_{c}z+g\left(z,\bar{z}\right), \end{array}$$ (35)

where

$$\begin{array}{c} g\left(z,\bar{z}\right)=\left(\Psi_{1}\left(0\right)-\text{i}\Psi_{2}\left(0\right)\right)\langle F\left(U_{t},0\right),f_{k}\rangle =\left(\Psi_{1}\left(0\right)-\text{i}\Psi_{2}\left(0\right)\right)\langle f\left(U_{t},0\right),f_{k}\rangle , \end{array}$$ (36)

and setting

$$\begin{array}{c} g\left(z,\bar{z}\right)=g_{20}\frac{z^{2}}{2}+g_{11}z\bar{z}+g_{02}\frac{{\bar{z}}^{2}}{2}+g_{21}\frac{z^{2}\bar{z}}{2}\ldots_{.} \end{array}$$ (37)

From *f*(*ϕ*, *a*) and Eq (31), it is easy to compute

$$\begin{array}{cc} \langle f\left(U_{t},0\right),f_{k}\rangle & =\frac{\tau_{c}}{4}\cdot \left(\begin{array}{c} 2f_{101}^{\left(1\right)}\xi e^{-\text{i}w_{k}\tau_{c}}+f_{002}^{\left(1\right)}\xi^{2}e^{-2\text{i}w_{k}\tau_{c}}\\ 2f_{101}^{\left(2\right)}\xi e^{-\text{i}w_{k}\tau_{c}}+f_{002}^{\left(2\right)}\xi^{2}e^{-2\text{i}w_{k}\tau_{c}} \end{array}\right)\cdot \frac{1}{\pi }\int_{0}^{\pi }{\left(\text{cos}kx\right)}^{3}dx\cdot \frac{z^{2}}{2}\\ & +\frac{\tau_{c}}{4}\cdot \left(\begin{array}{c} f_{101}^{\left(1\right)}\left(\bar{\xi }e^{\text{i}w_{k}\tau_{c}}+\xi e^{-\text{i}w_{k}\tau_{c}}\right)+f_{002}^{\left(1\right)}\xi \bar{\xi }\\ f_{101}^{\left(2\right)}\left(\bar{\xi }e^{\text{i}w_{k}\tau_{c}}+\xi e^{-\text{i}w_{k}\tau_{c}}\right)+f_{002}^{\left(2\right)}\xi \bar{\xi } \end{array}\right)\cdot \frac{1}{\pi }\int_{0}^{\pi }{\left(\text{cos}kx\right)}^{3}dx\cdot z\bar{z}\\ & +\frac{\tau_{c}}{4}\cdot \left(\begin{array}{c} 2f_{101}^{\left(1\right)}\bar{\xi }e^{\text{i}w_{k}\tau_{c}}+f_{002}^{\left(1\right)}{\bar{\xi }}^{2}e^{2\text{i}w_{k}\tau_{c}}\\ 2f_{101}^{\left(2\right)}\bar{\xi }e^{\text{i}w_{k}\tau_{c}}+f_{002}^{\left(2\right)}{\bar{\xi }}^{2}e^{2\text{i}w_{k}\tau_{c}} \end{array}\right)\cdot \frac{1}{\pi }\int_{0}^{\pi }{\left(\text{cos}kx\right)}^{3}dx\cdot \frac{{\bar{z}}^{2}}{2} \end{array}$$

$$\begin{array}{cc} & +\tau_{c}\cdot \left(\begin{array}{c} f_{101}^{\left(1\right)}\langle \left(W_{11}^{\left(2\right)}\left(-1\right)+\frac{1}{2}W_{20}^{\left(2\right)}\left(-1\right)\right)\cdot \text{cos}kx,\text{cos}kx\rangle \\ +f_{101}^{\left(1\right)}\langle \left(W_{11}^{\left(1\right)}\left(0\right)\xi e^{-\text{i}w_{k}\tau_{c}}+\frac{1}{2}W_{20}^{\left(1\right)}\left(0\right)\bar{\xi }e^{\text{i}w_{k}\tau_{c}}\right)\cdot \text{cos}kx,\text{cos}kx\rangle \\ +f_{002}^{\left(1\right)}\langle \left(W_{11}^{\left(2\right)}\left(-1\right)\xi e^{-\text{i}w_{k}\tau_{c}}+\frac{1}{2}W_{20}^{\left(2\right)}\left(-1\right)\bar{\xi }e^{\text{i}w_{k}\tau_{c}}\right)\cdot \text{cos}kx,\text{cos}kx\rangle \\ +\frac{1}{8}f_{102}^{\left(1\right)}\langle \left(2\xi \bar{\xi }+\xi^{2}e^{-2\text{i}w_{k}\tau_{c}}\right){\text{cos}}^{3}kx,\text{cos}kx\rangle \\ f_{101}^{\left(2\right)}\langle \left(W_{11}^{\left(2\right)}\left(-1\right)+\frac{1}{2}W_{20}^{\left(2\right)}\left(-1\right)\right)\cdot \text{cos}kx,\text{cos}kx\rangle \\ +f_{101}^{\left(2\right)}\langle \left(W_{11}^{\left(1\right)}\left(0\right)\xi e^{-\text{i}w_{k}\tau_{c}}+\frac{1}{2}W_{20}^{\left(1\right)}\left(0\right)\bar{\xi }e^{\text{i}w_{k}\tau_{c}}\right)\cdot \text{cos}kx,\text{cos}kx\rangle \\ +f_{002}^{\left(2\right)}\langle \left(W_{11}^{\left(2\right)}\left(-1\right)\xi e^{-\text{i}w_{k}\tau_{c}}+\frac{1}{2}W_{20}^{\left(2\right)}\left(-1\right)\bar{\xi }e^{\text{i}w_{k}\tau_{c}}\right)\cdot \text{cos}kx,\text{cos}kx\rangle \\ +\frac{1}{8}f_{102}^{\left(2\right)}\langle \left(2\xi \bar{\xi }+\xi^{2}e^{-2\text{i}w_{k}\tau_{c}}\right){\text{cos}}^{3}kx,\text{cos}kx\rangle \end{array}\right)\cdot \frac{z^{2}\bar{z}}{2}+..., \end{array}$$

where

$$\begin{array}{c} \langle W_{ij}^{\left(m\right)}\left(\theta \right),\text{cos}kx\rangle =\frac{1}{\pi }\int_{0}^{\pi }W_{ij}^{\left(m\right)}\left(\theta \right)\left(x\right)\text{cos}kxdx, \end{array}$$

and *i*, *j* = 0, 1, 2, …, *m* = 1, 2.

Let (*ψ*_1_, *ψ*_2_) = Ψ_1_−iΨ_2_, then we can obtain

$$\begin{array}{c} g_{20}=\left\{\begin{array}{cc} & \;\;\;\;\;0,\;\;\;\;\;\;\;\;\;\;\;\;\;\;\;\;\;k\in N,\\ & \frac{\tau_{c}}{4}\left[\left(2f_{101}^{\left(1\right)}\xi e^{-\text{i}w_{k}\tau_{c}}+f_{002}^{\left(1\right)}\xi^{2}e^{-2\text{i}w_{k}\tau_{c}}\right)\psi_{1}+\left(2f_{101}^{\left(2\right)}\xi e^{-\text{i}w_{k}\tau_{c}}+f_{002}^{\left(2\right)}\xi^{2}e^{-2\text{i}w_{k}\tau_{c}}\right)\psi_{2}\right],\;k=0, \end{array}\right. \end{array}$$

$$\begin{array}{c} g_{11}=\left\{\begin{array}{cc} & \;\;\;\;\;0,\;\;\;\;\;\;\;\;\;\;\;\;\;\;\;\;\;k\in N,\\ & \frac{\tau_{c}}{4}\left\{\left[f_{101}^{\left(1\right)}\left(\bar{\xi }e^{\text{i}w_{k}\tau_{c}}+\xi e^{-\text{i}w_{k}\tau_{c}}\right)+f_{002}^{\left(1\right)}\xi \bar{\xi }\right]\psi_{1}+\left[f_{101}^{\left(2\right)}\left(\bar{\xi }e^{\text{i}w_{k}\tau_{c}}+\xi e^{-\text{i}w_{k}\tau_{c}}\right)+f_{002}^{\left(2\right)}\xi \bar{\xi }\right]\psi_{2}\right\},\;k=0, \end{array}\right. \end{array}$$

$$\begin{array}{c} g_{02}=\bar{g_{20}}, \end{array}$$

$$\begin{array}{cc} g_{21} & =\tau_{c}\left(\begin{array}{c} f_{101}^{\left(1\right)}\langle \left(W_{11}^{\left(2\right)}\left(-1\right)+\frac{1}{2}W_{20}^{\left(2\right)}\left(-1\right)\right)\cdot \text{cos}kx,\text{cos}kx\rangle \\ +f_{101}^{\left(1\right)}\langle \left(W_{11}^{\left(1\right)}\left(0\right)\xi e^{-\text{i}w_{k}\tau_{c}}+\frac{1}{2}W_{20}^{\left(1\right)}\left(0\right)\bar{\xi }e^{\text{i}w_{k}\tau_{c}}\right)\cdot \text{cos}kx,\text{cos}kx\rangle \\ +f_{002}^{\left(1\right)}\langle \left(W_{11}^{\left(2\right)}\left(-1\right)\xi e^{-\text{i}w_{k}\tau_{c}}+\frac{1}{2}W_{20}^{\left(2\right)}\left(-1\right)\bar{\xi }e^{\text{i}w_{k}\tau_{c}}\right)\cdot \text{cos}kx,\text{cos}kx\rangle \\ +\frac{1}{8}f_{102}^{\left(1\right)}\langle \left(2\xi \bar{\xi }+\xi^{2}e^{-2\text{i}w_{k}\tau_{c}}\right){\text{cos}}^{3}kx,\text{cos}kx\rangle \end{array}\right)\psi_{1}\\ & +\tau_{c}\left(\begin{array}{c} f_{101}^{\left(2\right)}\langle \left(W_{11}^{\left(2\right)}\left(-1\right)+\frac{1}{2}W_{20}^{\left(2\right)}\left(-1\right)\right)\cdot \text{cos}kx,\text{cos}kx\rangle \\ +f_{101}^{\left(2\right)}\langle \left(W_{11}^{\left(1\right)}\left(0\right)\xi e^{-\text{i}w_{k}\tau_{c}}+\frac{1}{2}W_{20}^{\left(1\right)}\left(0\right)\bar{\xi }e^{\text{i}w_{k}\tau_{c}}\right)\cdot \text{cos}kx,\text{cos}kx\rangle \\ +f_{002}^{\left(2\right)}\langle \left(W_{11}^{\left(2\right)}\left(-1\right)\xi e^{-\text{i}w_{k}\tau_{c}}+\frac{1}{2}W_{20}^{\left(2\right)}\left(-1\right)\bar{\xi }e^{\text{i}w_{k}\tau_{c}}\right)\cdot \text{cos}kx,\text{cos}kx\rangle \\ +\frac{1}{8}f_{102}^{\left(2\right)}\langle \left(2\xi \bar{\xi }+\xi^{2}e^{-2\text{i}w_{k}\tau_{c}}\right){\text{cos}}^{3}kx,\text{cos}kx\rangle \end{array}\right)\psi_{2},\;k\in \left\{0,N\right\}. \end{array}$$

Since the expression of *g*_21_ containing *W*_20_(*θ*) and *W*_11_(*θ*) for *θ* ∈ [−1, 0], it is necessary to compute them. From Eq (34), we can derive

$$\begin{array}{c} \dot{W}\left(z,\bar{z}\right)=W_{20}z\dot{z}+W_{11}\dot{z}\bar{z}+W_{11}z\dot{\bar{z}}+W_{02}\bar{z}\dot{\bar{z}}+..., \end{array}$$ (38)

$$\begin{array}{c} A\left(\tau_{c}\right)W=A\left(\tau_{c}\right)W_{20}\frac{z^{2}}{2}+A\left(\tau_{c}\right)W_{11}z\bar{z}+A\left(\tau_{c}\right)W_{02}\frac{{\bar{z}}^{2}}{2}. \end{array}$$ (39)

Meanwhile, from the conclusion of literature [42],

$$\begin{array}{c} \dot{W}=A\left(\tau_{c}\right)W+H\left(z,\bar{z}\right), \end{array}$$ (40)

where

$$\begin{array}{c} H\left(z,\bar{z}\right)=H_{20}\frac{z^{2}}{2}+H_{11}z\bar{z}+H_{02}\frac{{\bar{z}}^{2}}{2}+...=X_{0}f\left(U_{t},0\right)-\Phi \left(\Psi ,\langle X_{0}f\left(U_{t},0\right),f_{k}\rangle \right)\cdot f_{k}, \end{array}$$ (41)

with *H*_*ij*_ ∈ P*, *i*, *j* = 0, 1, 2….

Therefore, from Eqs (35) and (37)–(41), the following form can be given by:

$$\begin{array}{c} \left\{\begin{array}{cc} & \left(2\text{i}w_{k}\tau_{c}-A\left(\tau_{c}\right)\right)W_{20}=H_{20},\\ & -A\left(\tau_{c}\right)W_{11}=H_{11}. \end{array}\right. \end{array}$$ (42)

Because *A*(*τ*_*c*_) has only two eigenvalues ±i*w*_*k*_ *τ*_*c*_, Eq (42) has unique solution *W*_*ij*_ in the following form:

$$\begin{array}{c} \left\{\begin{array}{cc} & W_{20}={\left(2\text{i}w_{k}\tau_{c}-A\left(\tau_{c}\right)\right)}^{-1}H_{20},\\ & W_{11}=-A{\left(\tau_{c}\right)}^{-1}H_{11}. \end{array}\right. \end{array}$$ (43)

From Eq (41), for −1 ≤ *θ* < 0,

$$\begin{array}{ll} H(z,\bar{z}) & =-\Phi (\theta )\Psi (0)\langle f(U_{t},0),f_{k}\rangle \cdot f_{k}\\ & =-(\frac{q_{1}(\theta )+q_{2}(\theta )}{2},\frac{q_{1}(\theta )-q_{2}(\theta )}{2\text{i}})(\Psi_{1}(0),\Psi_{2}(0))\cdot \langle f(U_{t},0),f_{k}\rangle \cdot f_{k}\\ & =-\frac{1}{2}(q_{1}(\theta )g_{20}+q_{2}(\theta )\bar{g_{02}})\cdot f_{k}\frac{z^{2}}{2}-\frac{1}{2}(q_{1}(\theta )g_{11}+q_{2}(\theta )\bar{g_{11}})\cdot f_{k}z\bar{z}+\ldots_{.} \end{array}$$

Thus, for −1 ≤ *θ* < 0,

$$\begin{array}{c} H_{20}\left(\theta \right)=\left\{\begin{array}{cc} & \;\;\;\;\;0,\;\;\;\;\;\;\;\;k\in N,\\ & -\frac{1}{2}\left(q_{1}\left(\theta \right)g_{20}+q_{2}\left(\theta \right)\bar{g_{02}}\right)\cdot f_{0},\;k=0, \end{array}\right. \end{array}$$ (44)

$$\begin{array}{c} H_{11}\left(\theta \right)=\left\{\begin{array}{cc} & \;\;\;\;\;0,\;\;\;\;\;\;\;\;k\in N,\\ & -\frac{1}{2}\left(q_{1}\left(\theta \right)g_{11}+q_{2}\left(\theta \right)\bar{g_{11}}\right)\cdot f_{0},\;k=0. \end{array}\right. \end{array}$$ (45)

For *θ* = 0, $H\left(z,\bar{z}\right)\left(0\right)=f\left(U_{t},0\right)-\Phi \left(\Psi ,\langle f\left(U_{t},0\right),f_{k}\rangle \right)\cdot f_{k}$, we have

$$\begin{array}{c} H_{20}\left(0\right)=\left\{\begin{array}{cc} & \frac{\tau_{c}}{4}\left(\begin{array}{c} 2f_{101}^{\left(1\right)}\xi e^{-\text{i}w_{k}\tau_{c}}+f_{002}^{\left(1\right)}\xi^{2}e^{-2\text{i}w_{k}\tau_{c}}\\ 2f_{101}^{\left(2\right)}\xi e^{-\text{i}w_{k}\tau_{c}}+f_{002}^{\left(2\right)}\xi^{2}e^{-2\text{i}w_{k}\tau_{c}} \end{array}\right)\cdot {\left(\text{cos}kx\right)}^{2},\;\;\;\;\;\;\;\;\;k\in N,\\ & \frac{\tau_{c}}{4}\left(\begin{array}{c} 2f_{101}^{\left(1\right)}\xi e^{-\text{i}w_{k}\tau_{c}}+f_{002}^{\left(1\right)}\xi^{2}e^{-2\text{i}w_{k}\tau_{c}}\\ 2f_{101}^{\left(2\right)}\xi e^{-\text{i}w_{k}\tau_{c}}+f_{002}^{\left(2\right)}\xi^{2}e^{-2\text{i}w_{k}\tau_{c}} \end{array}\right)-\frac{1}{2}\left(q_{1}\left(0\right)g_{20}+q_{2}\left(0\right)\bar{g_{02}}\right)\cdot f_{0},\;k=0, \end{array}\right. \end{array}$$ (46)

$$\begin{array}{c} H_{11}\left(0\right)=\left\{\begin{array}{cc} & \frac{\tau_{c}}{4}\left(\begin{array}{c} f_{101}^{\left(1\right)}\left(\bar{\xi }e^{\text{i}w_{k}\tau_{c}}+\xi e^{-\text{i}w_{k}\tau_{c}}\right)+f_{002}^{\left(1\right)}\xi \bar{\xi }\\ f_{101}^{\left(2\right)}\left(\bar{\xi }e^{\text{i}w_{k}\tau_{c}}+\xi e^{-\text{i}w_{k}\tau_{c}}\right)+f_{002}^{\left(2\right)}\xi \bar{\xi } \end{array}\right)\cdot {\left(\text{cos}kx\right)}^{2},\;\;\;\;\;\;\;\;\;k\in N,\\ & \frac{\tau_{c}}{4}\left(\begin{array}{c} f_{101}^{\left(1\right)}\left(\bar{\xi }e^{\text{i}w_{k}\tau_{c}}+\xi e^{-\text{i}w_{k}\tau_{c}}\right)+f_{002}^{\left(1\right)}\xi \bar{\xi }\\ f_{101}^{\left(2\right)}\left(\bar{\xi }e^{\text{i}w_{k}\tau_{c}}+\xi e^{-\text{i}w_{k}\tau_{c}}\right)+f_{002}^{\left(2\right)}\xi \bar{\xi } \end{array}\right)-\frac{1}{2}\left(q_{1}\left(0\right)g_{11}+q_{2}\left(0\right)\bar{g_{11}}\right)\cdot f_{0},\;k=0. \end{array}\right. \end{array}$$ (47)

Based on the definition of infinitesimal generator *A*(*τ*_*c*_), then Eq (42) is transformed into

$$\begin{array}{c} {\dot{W}}_{20}\left(\theta \right)=2\text{i}w_{k}\tau_{c}W_{20}\left(\theta \right)+\frac{1}{2}\left(q_{1}\left(\theta \right)g_{20}+q_{2}\left(\theta \right)\bar{g_{02}}\right)\cdot f_{0}, \end{array}$$ (48)

and −1 ≤ *θ* < 0.

From *q*_1_(*θ*) = *q*_1_(0)*e*^i*w*_*k*_*τ*_*c*_*θ*^, − 1 ≤ *θ* ≤ 0, we have

$$\begin{array}{c} W_{20}\left(\theta \right)=\frac{1}{2}\left[\frac{\text{i}g_{20}}{w_{k}\tau_{c}}q_{1}\left(\theta \right)+\frac{\text{i}\bar{g_{02}}}{3w_{k}\tau_{c}}q_{2}\left(\theta \right)\right]\cdot f_{k}+C_{1}e^{2\text{i}w_{k}\tau_{c}\theta }, \end{array}$$ (49)

further we obtain

$$\begin{array}{c} C_{1}=\left\{\begin{array}{cc} & \;\;\;\;\;\;W_{20}\left(0\right),\;\;\;\;\;\;\;\;\;\;k\in N,\\ & W_{20}\left(0\right)-\frac{1}{2}\left[\frac{\text{i}g_{20}}{w_{k}\tau_{c}}q_{1}\left(0\right)+\frac{\text{i}\bar{g_{02}}}{3w_{k}\tau_{c}}q_{2}\left(0\right)\right]\cdot f_{0},\;k=0. \end{array}\right. \end{array}$$ (50)

For *k* = 0, *θ* = 0, in the light of the definition of *A*(*τ*_*c*_) and Eq (49), the first Eq of Eq (42) becomes

$$\begin{array}{c} 2\text{i}w_{k}\tau_{c}\left[\frac{1}{2}\left(\frac{\text{i}g_{20}}{w_{k}\tau_{c}}q_{1}\left(0\right)+\frac{\text{i}\bar{g_{02}}}{3w_{k}\tau_{c}}q_{2}\left(0\right)\right)\cdot f_{0}+C_{1}\right]\\ -\tau_{c}D\frac{\partial^{2}}{\partial x^{2}}\left[\frac{1}{2}\left(\frac{\text{i}g_{20}}{w_{k}\tau_{c}}q_{1}\left(0\right)+\frac{\text{i}\bar{g_{02}}}{3w_{k}\tau_{c}}q_{2}\left(0\right)\right)\cdot f_{0}+C_{1}\right]\\ -L\left(\tau_{c}\right)\left[\frac{1}{2}\left(\frac{\text{i}g_{20}}{w_{k}\tau_{c}}q_{1}\left(\theta \right)+\frac{\text{i}\bar{g_{02}}}{3w_{k}\tau_{c}}q_{2}\left(\theta \right)\right)\cdot f_{0}+C_{1}e^{2\text{i}w_{k}\tau_{c}\theta }\right]\\ =\frac{\tau_{c}}{4}\left(\begin{array}{c} 2f_{101}^{\left(1\right)}\xi e^{-\text{i}w_{k}\tau_{c}}+f_{002}^{\left(1\right)}\xi^{2}e^{-2\text{i}w_{k}\tau_{c}}\\ 2f_{101}^{\left(2\right)}\xi e^{-\text{i}w_{k}\tau_{c}}+f_{002}^{\left(2\right)}\xi^{2}e^{-2\text{i}w_{k}\tau_{c}} \end{array}\right)-\frac{1}{2}\left(q_{1}\left(0\right)g_{20}+q_{2}\left(0\right)\bar{g_{02}}\right)\cdot f_{0}. \end{array}$$

So we can derive

$$\begin{array}{c} 2\text{i}w_{k}\tau_{c}C_{1}-\tau_{c}D\frac{\partial^{2}}{\partial x^{2}}C_{1}-L\left(\tau_{c}\right)\left(C_{1}e^{2\text{i}w_{k}\tau_{c}\theta }\right)=\frac{\tau_{c}}{4}\left(\begin{array}{c} 2f_{101}^{\left(1\right)}\xi e^{-\text{i}w_{k}\tau_{c}}+f_{002}^{\left(1\right)}\xi^{2}e^{-2\text{i}w_{k}\tau_{c}}\\ 2f_{101}^{\left(2\right)}\xi e^{-\text{i}w_{k}\tau_{c}}+f_{002}^{\left(2\right)}\xi^{2}e^{-2\text{i}w_{k}\tau_{c}} \end{array}\right){\left(\text{cos}kx\right)}^{2}. \end{array}$$ (51)

From Eq (51), the formula of *C*_1_ can be derived

$$\begin{array}{cc} C_{1}= & \frac{1}{4}{\left(\begin{array}{cc} 2\text{i}w_{k}+d_{1}k^{2}-a_{11} & -a_{13}e^{-2\text{i}w_{k}\tau_{c}}\\ -a_{21} & 2\text{i}w_{k}+d_{2}k^{2}-a_{22}-a_{23}e^{-2\text{i}w_{k}\tau_{c}} \end{array}\right)}^{-1}J_{1}, \end{array}$$ (52)

where

$$\begin{array}{c} J_{1}=\left(\begin{array}{c} 2f_{101}^{\left(1\right)}\xi e^{-\text{i}w_{k}\tau_{c}}+f_{002}^{\left(1\right)}\xi^{2}e^{-2\text{i}w_{k}\tau_{c}}\\ 2f_{101}^{\left(2\right)}\xi e^{-\text{i}w_{k}\tau_{c}}+f_{002}^{\left(2\right)}\xi^{2}e^{-2\text{i}w_{k}\tau_{c}} \end{array}\right){\left(\text{cos}kx\right)}^{2}. \end{array}$$

For −1 ≤ *θ* < 0, similar to the above case, *W*_11_(*θ*) can be obtained

$$\begin{array}{c} {\dot{W}}_{11}\left(\theta \right)=\frac{1}{2}\left(q_{1}\left(\theta \right)g_{11}+q_{2}\left(\theta \right)\bar{g_{11}}\right)\cdot f_{0}, \end{array}$$ (53)

$$\begin{array}{c} W_{11}\left(\theta \right)=\frac{1}{2}\left(\frac{-\text{i}g_{11}}{w_{k}\tau_{c}}q_{1}\left(\theta \right)+\frac{\text{i}\bar{g_{11}}}{w_{k}\tau_{c}}q_{2}\left(\theta \right)\right)\cdot f_{k}+C_{2}, \end{array}$$ (54)

further we derive

$$\begin{array}{c} C_{2}=\frac{1}{4}{\left(\begin{array}{cc} d_{1}k^{2}-a_{11} & -a_{13}\\ -a_{21} & d_{2}k^{2}-a_{22}-a_{23} \end{array}\right)}^{-1}J_{2}, \end{array}$$ (55)

where

$$\begin{array}{c} J_{2}=\left(\begin{array}{c} f_{101}^{\left(1\right)}\left(\bar{\xi }e^{\text{i}w_{k}\tau_{c}}+\xi e^{-\text{i}w_{k}\tau_{c}}\right)+f_{002}^{\left(1\right)}\xi \bar{\xi }\\ f_{101}^{\left(2\right)}\left(\bar{\xi }e^{\text{i}w_{k}\tau_{c}}+\xi e^{-\text{i}w_{k}\tau_{c}}\right)+f_{002}^{\left(2\right)}\xi \bar{\xi } \end{array}\right){\left(\text{cos}kx\right)}^{2}. \end{array}$$

Through the above calculations of *W*_20_(*θ*) and *W*_11_(*θ*), we obtain the expression of *g*_21_. Consequently, in order to determine the properties of Hopf bifurcating period solutions at the critical value *τ*_*c*_, we can compute the following values:

$$\begin{array}{c} c_{1}\left(0\right)=\frac{\text{i}}{2w_{k}\tau_{c}}\left(g_{20}g_{11}-2|g_{11}{|}^{2}-\frac{1}{3}{\left|g_{02}\right|}^{2}\right)+\frac{1}{2}g_{21},\\ \mu_{2}=-\frac{\text{Re}(c_{1}\left(0\right))}{\text{Re}(\lambda^{{}^{\prime }}\left(\tau_{k}^{j}\right))},\\ \beta_{2}=2\text{Re}\left(c_{1}\left(0\right)\right),\\ T_{2}=-\frac{1}{w_{k}\tau_{c}}\left(\text{Im}\left(c_{1}\left(0\right)\right)+\mu_{2}\text{Im}\left(\lambda^{{}^{\prime }}\left(\tau_{k}^{j}\right)\right)\right). \end{array}$$ (56)

*μ*_2_ > 0 (*μ*_2_ < 0) determines the direction of the Hopf bifurcation is supercritical (*τ* > *τ*_*c*_) (subcritical (*τ* < *τ*_*c*_)); if *β*_2_ < 0 (*β*_2_ > 0) indicates that the bifurcating period solutions on center manifold are asymptotically stable (unstable); furthermore, *T*_2_ can determine the period of the bifurcating period solutions, namely, *T*_2_ < 0 (*T*_2_ > 0) represents the decrease (increase) of the period.

### Numerical results

Compared with the theoretical analyses, we perform a series of extensive numerical simulations of the spatiotemporal epidemic model with nonlinear incidence rate in one-dimensional space, and investigate the incubation period how to affect the spread of epidemics. We solve the numerical solutions of system (1) by using Matlab. The reaction-diffusion system is solved in a discrete domain with *N*_*x*_ × *N*_*y*_ lattice sites. The Laplacian describing diffusion is approximated by using finite differences, and we also discretize the time evolution.

**Fig 1:**
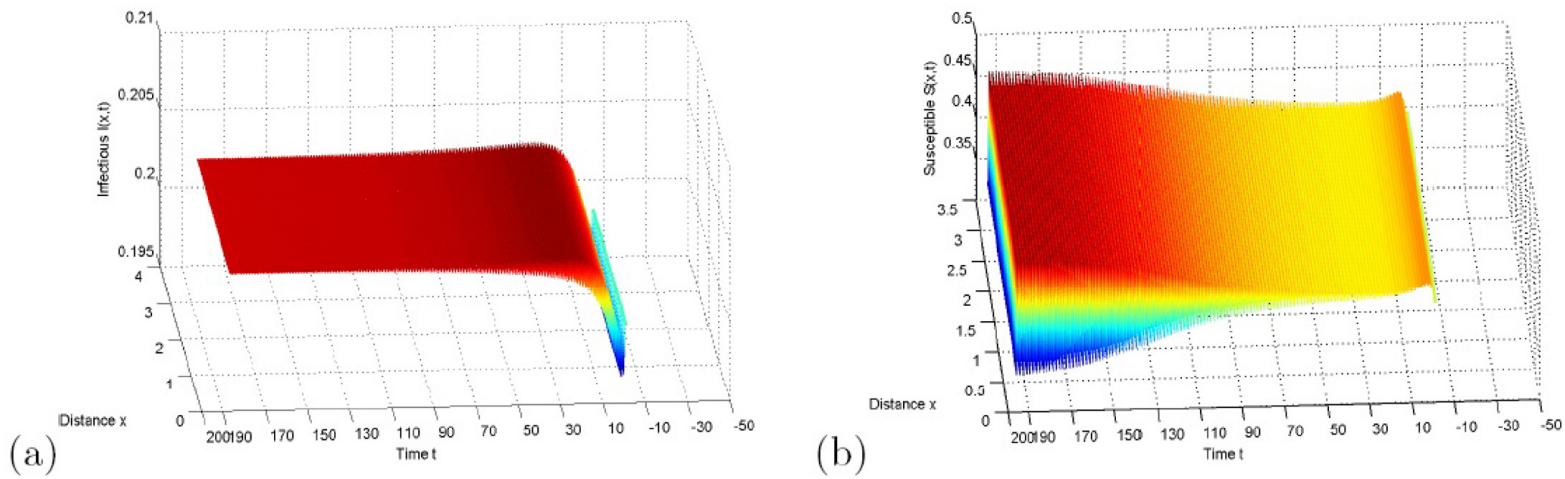
Hopf bifurcation with *k* = 0. (a) The constant steady state E* is asymptotically stable for *τ* = 1.2 with initial conditions *S*(*x*, *t*) = 0.42, *I*(*x*, *t*) = 0.20, (*x*, *t*) ∈ [0, *π*] × [−1.2, 0]; (b) The bifurcating periodic solutions are asymptotically stable for *τ* = 1.6 with initial conditions *S*(*x*, *t*) = 0.42, *I*(*x*, *t*) = 0.20, (*x*, *t*) ∈ [0, *π*] × [−1.6, 0].

In case *k* = 0, we set *d*_1_ = 6, *d*_2_ = 1, *A* = 1, *β* = 32, *μ* = 1.8, *d* = 1, the equilibrium is E* = (S*, I*) = (0.43, 0.20). By some calculations, $\tau_{0}^{0}=1.33$, *c*_1_(0) = −9.81 + 22.15i are obtained. Through the formulae of properties of Hopf bifurcating period solutions in section III, we get *μ*_2_ > 0, *β*_2_ < 0 and *T*_2_ > 0. These parameter values shows E* is asymptotically stable for 0 ≤ *τ* < *τ*_*c*_. With the increase of *τ*, E* loses its stability and Hopf bifurcation occurs at critical point *τ*_*c*_, these bifurcating period solutions are stable, the direction of bifurcation is forward and the period increases, which are presented in Fig 1.

**Fig 2:**
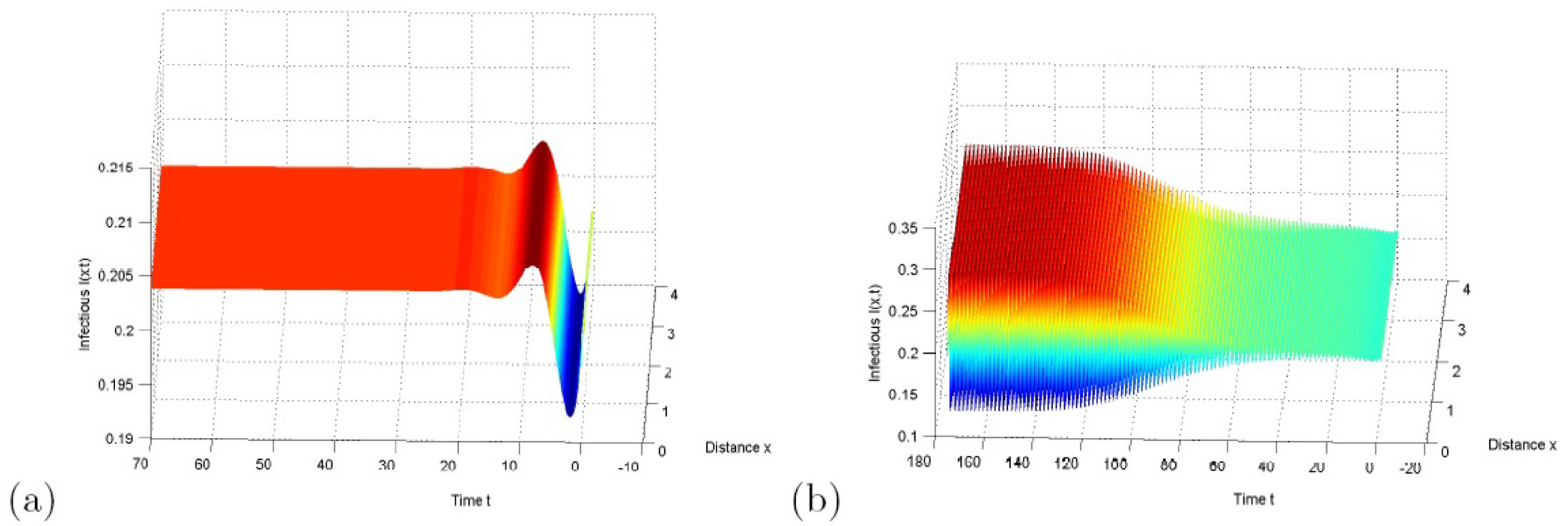
Hopf bifurcation with *k* = 1. (a) When *τ* = 0.3, the constant steady state E* is asymptotically stable with initial conditions *S*(*x*, *t*) = 0.42, *I*(*x*, *t*) = 0.20, (*x*, *t*) ∈ [0, *π*] × [−0.3, 0]; (b) When *τ* = 1.5, the bifurcating periodic solutions are asymptotically stable with initial conditions *S*(*x*, *t*) = 0.42, *I*(*x*, *t*) = 0.20, (*x*, *t*) ∈ [0, *π*] × [−1.5, 0].

In case *k* = 1, setting *d*_1_ = 6, *d*_2_ = 1, *A* = 1, *β* = 32, *μ* = 1.8, *d* = 1, then the equilibrium is E* = (S*, I*) = (0.43, 0.20). Furthermore, by using the formulae derived in section III, we compute $\tau_{1}^{0}=0.45$, *c*_1_(0) = 2.30 × 10^2^ − 7.55 × 10^1^i. By computing the formulae (58), *μ*_2_ > 0, *β*_2_ < 0 and *T*_2_ < 0 are obtained, which indicates that these bifurcating period solutions are stable, the direction of Hopf bifurcation is forward, and the period decreases. These phenomena are showed in Fig 2.

## Discussion

In this study, the characteristic equation at the positive constant steady state E*(S*, I*) is derived. In order to study the influence of incubation period on epidemic transmission, we choose time delay *τ* as a bifurcation parameter. Moreover, we get the two classes conditions of the existence of Hopf bifurcation: one is the absence of diffusion *k* = 0, the other is the presence of diffusion *k* = *k*_0_ ∈ *N*. With increasing of parameter *τ*, the stability of positive constant steady state E*(S*, I*) will change, and Hopf bifurcation will concurrently occur in system (1) at the critical point *τ*_*c*_($\tau_{0}^{j}$ or $\tau_{k_{0}}^{j}$). In the following, we obtain the properties of bifurcating period solutions including direction, stability and period by utilizing the normal formal theory and the center manifold theorem of partial functional differential equations (PFDs).

It should be noted that spatial pattern may be found in epidemic model (1). Based on pattern dynamics of model (1), one can obtain the pattern structures in different parameters space [44, 45]. In this case, we can reveal the distributions of disease with high density or low density and thus provide useful control measures to eliminate the disease.

## Conclusion

The numerical results validate our theoretical findings, which show that the length of the incubation period have significant impacts on epidemic transmission. The biennial outbreaks of measles is the signature of an endemic infectious disease, which becomes non-endemic if there were a minor increase in infectivity or a decrease in the length of the incubation period [15]. Based on this paper, we provide a possible mechanism to explain the recurrent outbreak of disease.

## Supporting Information

S1 Fig Hopf bifurcation with *k* = 0. (a) The constant steady state E* is asymptotically stable for *τ* = 1.2 with initial conditions *S*(*x*, *t*) = 0.42, *I*(*x*, *t*) = 0.20, (*x*, *t*) ∈ [0, *π*] × [−1.2, 0]; (b) The bifurcating periodic solutions are asymptotically stable for *τ* = 1.6 with initial conditions *S*(*x*, *t*) = 0.42, *I*(*x*, *t*) = 0.20, (*x*, *t*) ∈ [0, *π*] × [−1.6, 0].
(EPS)

S2 Fig Hopf bifurcation with *k* = 1. (a) When *τ* = 0.3, the constant steady state E* is asymptotically stable with initial conditions *S*(*x*, *t*) = 0.42, *I*(*x*, *t*) = 0.20, (*x*, *t*) ∈ [0, *π*] × [−0.3, 0]; (b) When *τ* = 1.5, the bifurcating periodic solutions are asymptotically stable with initial conditions *S*(*x*, *t*) = 0.42, *I*(*x*, *t*) = 0.20, (*x*, *t*) ∈ [0, *π*] × [−1.5, 0].
(EPS)

S1 File Transversality condition. The relationship between real part of eigenvalues and time delay.
(PDF)

## References

[1] Soper HE. The interpretation of periodicity in disease prevalence. J. R. Stat. Soc. 1929; 92: 34–73. 10.2307/2341437

[2] Song LP, Jin Z, Sun GQ. Reinfection induced disease in a spatial SIRI model. Journal of Biological Physics 2011; 37: 133–140. 10.1007/s10867-010-9204-6 22210967 PMC3006469

[3] Hoppensteadt F, Waltman P. A problem in the theory of epidemics. II, Math. Biosci. 1971; 12: 133–146.

[4] Bnrtlett MS. Deterministic and stochastic models for recurrent epidemics, Proceedings of the Third Berkeley Symposium on Mathematical Statistics and Probability, Berkeley and Los Angeles, University of California Press 1956; 4: 81–109.

[5] Maines TR, Jayaraman A, Belser JA, Wadford DA, Pappas C, Zeng H, et al Transmission and pathogenesis of swine-origin 2009 A (H1N1) influenza viruses in ferrets and mice. Science 2009; 325: 484–487. 10.1126/science.1177238 19574347 PMC2953552

[6] Zhang J, Jin Z, Sun GQ, Sun XD, Ruan S. Modeling seasonal rabies epidemics in China. Bull. Math. Biol. 2012; 74: 1226–1251. 10.1007/s11538-012-9720-6 22383117 PMC7089220

[7] Dye C, Gay N. Modeling the SARS epidemic. Science 2003; 300: 1884–1885. 10.1126/science.1086925 12766208

[8] Team WER. Ebola virus disease in West Africa-the first 9 months of the epidemic and forward projections. N. Engl. J. Med. 2014; 371: 1481–95. 10.1056/NEJMoa1411100 25244186 PMC4235004

[9] Sun GQ, Wu ZY, Wang Z, Jin Z. Influence of isolation degree of spatial patterns on persistence of populations. Nonlinear Dynamics 2016; 83: 811–819. 10.1007/s11071-015-2369-6

[10] Huo X, Shi G, Li X, Deng L, Xu F, Chen M, et al Knowledge and attitudes about Ebola vaccine among the general population in Sierra Leone. Vaccine 2016; 34: 1767–1772. 10.1016/j.vaccine.2016.02.046 26928073

[11] Rizzo A, Pedalino B, Porfiri M. A network model for ebola spreading. J. Theoret. Biol. 2016; 394: 212–222. 10.1016/j.jtbi.2016.01.015 26804645

[12] Xia ZQ, Wang SF, Li SL, Huang LY, Zhang WY, Sun GQ, et al Modeling the transmission dynamics of Ebola virus disease in Liberia. Sci. Rep. 2015; 5: 13857 10.1038/srep13857 26347015 PMC4561958

[13] Levin SA, Hallm TG, Gross LG. Applied mathematical ecology. Springer, New York, 1989.

[14] Capasso V. Mathematical structure of epidemic systems, in: Lecture Notes in Biomathematics. Springer, Berlin, 1993.

[15] London WP, Yorke JA. Recurrent outbreaks of measles, chickenpox and mumps I. Seasonal variation in contact rates. American journal of epidemiology 1973; 98: 453–468. 4767622 10.1093/oxfordjournals.aje.a121575

[16] Xiao DM, Ruan SG. Global analysis of an epidemic model with nonmonotone incidence rate. Math. Biosci. 2007; 208: 419–429. 10.1016/j.mbs.2006.09.025 17303186 PMC7094627

[17] Korobeinikov A. Global properties of infectious disease models with nonlinear incidence. Bull. Math. Biol. 2007; 69: 1871–1886. 10.1007/s11538-007-9196-y 17443392

[18] Capasso V, Serio G. A generalization of the Kermack-Mckendrick deterministic epidemic model. Math. Biosci. 1978; 42: 43–61. 10.1016/0025-5564(78)90006-8

[19] Anderson RM, May RM. Regulation and stability of host-parasite population interactions: I. Regulatory processes. J. Anim. Ecol. 1978; 47: 219–267. 10.2307/3933

[20] Li L. Patch invasion in a spatial epidemic model. Applied Mathematics and Computation 2015; 258: 342–349. 10.1016/j.amc.2015.02.006

[21] Sun GQ, Zhang J, Song LP, Jin Z, Li BL. Pattern formation of a spatial predator-prey system. Applied Mathematics and Computation 2012; 218: 11151–11162. 10.1016/j.amc.2012.04.071

[22] Beretta E, Takeuchi Y. Global stability of an SIR epidemic model with time delays. J. Math. Biol. 1995; 33: 250–260. 10.1007/BF00169563 7897328

[23] Kadder A. On the dyanmics of a delayed SIR epiemic model with a modified saturated incidence rate. Electronic J.D.E. 2009; 1: 1–7.

[24] Zhang J, Jin Z, Yan J, Sun G. Stability and Hopf bifurcation in a delayed competition system. Nonlinear Anal.: T.M.A. 2009; 70: 658–670. 10.1016/j.na.2008.01.002

[25] Abta A, Kadder A, Alaoui TH. Global stability for delay SIR and SEIR epidemic models with saturated incidece rates. Electronic J.D.E. 2012; 2012: 1–13.

[26] Li MT, Sun GQ, Wu YF, Zhang J, Jin Z. Transmission dynamics of a multi-group brucellosis model with mixed cross infection in public farm. Applied Mathematics and Computation 2014; 237: 582–594. 10.1016/j.amc.2014.03.094

[27] Sun GQ, Zhang ZK. Global stability for a sheep brucellosis model with immigration. Applied Mathematics and Computation 2014; 246: 336–345. 10.1016/j.amc.2014.08.028

[28] Sun GQ, Wang SL, Ren Q, Wu YP, Jin Z. Effects of time delay and space on herbivore dynamics: linking inducible defenses of plants to herbivore outbreak. Sci. Rep. 2015; 5: 11246 10.1038/srep11246 26084812 PMC4471659

[29] Li L, Jin Z, Li J. Periodic solutions in a herbivore-plant system with time delay and spatial diffusion. Applied Mathematical Modelling 2016; 40: 4765–4777. 10.1016/j.apm.2015.12.003

[30] Zhang J, Jin Z, Sun GQ, Sun X, Ruan S. Spatial spread of rabies in China. J. Appl. Analy. Compu. 2012; 2: 111–126.

[31] McCluskey CC. Global stability for an SIR epidemic model with delay and nonlinear incidence. Nonlinear Analysis: Real World Applications 2010; 11: 3106–3109. 10.1016/j.nonrwa.2009.11.005

[32] Sun C, Lin Y, Han M. Stability and Hopf bifurcation for an epidemic disease model with delay. Chaos, Solitons and Fractals 2006; 30: 204–216. 10.1016/j.chaos.2005.08.167

[33] Liu WM, Levin SA, Iwasa Y. Influence of nonlinear incidence rates upon the behavior of SIRS epidemiological models. J. Math. Biol. 1986; 23: 187–204. 10.1007/BF00276956 3958634

[34] Liu WM, Hethcote HW, Levin SA. Dynamical behavior of epidemiological models with nonlinear incidence rates. J. Math. Biol. 1987; 25: 359–380. 10.1007/BF00277162 3668394

[35] Busenberg S, Cooke KL. The effect of integral conditions in certain equations modelling epidmic and population growth. J. Math. Biol. 1980; 10: 13–32. 10.1007/BF00276393 7205075

[36] Klinkenberg D, Nishiura H. The correlation between infectivity and incubation period of measles, estimated from households with two cases. J. Theor. Biol. 2011; 284: 52–60. 10.1016/j.jtbi.2011.06.015 21704640

[37] White MC, Nelson RM, Kawamura LM, Grinsdale J, Goldenson J. Changes in characteristics of inmates with latent tuberculosis infection. Public Health 2012; 126: 752–759. 10.1016/j.puhe.2012.04.009 22840442

[38] Sun GQ. Pattern formation of an epidemic model with diffusion. Nonlinear Dynam. 2012; 69: 1097–1104. 10.1007/s11071-012-0330-5 PMC7088525 32214667

[39] Yan XP. Stability and Hopf bifurcation for a delayed prey-predator system with diffusion effects. Applied Mathematics and Computation 2007; 192: 552–556. 10.1016/j.amc.2007.03.033

[40] Zuo W, Wei J. Stability and Hopf bifurcation in a diffusive predator-prey system with delay effect. Nonlinear Anal. R.W.A. 2011; 12: 1998–2011. 10.1016/j.nonrwa.2010.12.016

[41] Faria T, Magalhaes LT. Normal form for retared functional differential equations with parameters and applications to Hopf bifurcation. J. Differential Equations 1995; 22: 181–200. 10.1006/jdeq.1995.1144

[42] Wu J. Theory and Applications of Partial Functional Differential Equations. Springer-Verlag, New York, 1996.

[43] Hale J. Theory of Functional Differential Equations. Springer-Verlag, Berlin, 1977.

[44] Sun G, Jin Z, Liu QX, Li L. Pattern formation in a S-I model with nonlinear incidence rates. J. Stat. Mech. 2007; 11: P11011 10.1088/1742-5468/2007/11/P11011

[45] Sun GQ, Jin Z, Liu QX, Li L. Chaos induced by breakup of waves in a spatial epidemic model with nonlinear incidence rate. J. Stat. Mech. 2008; 8: P08011.

