# Supplementary material for: Hopf Bifurcation of an Epidemic Model with Delay
Source: PLoS One. 2016 Jun 15;11(6):e0157367. doi: 10.1371/journal.pone.0157367 (PMC4909215; doi:10.1371/journal.pone.0157367)
Supplement: S1 File — The relationship between real part of eigenvalues and time delay. (PDF) [file pone.0157367.s003.pdf]

Hopf bifurcation of an epidemic model with delay

Li-Peng Song[1], Xiao-Qiang Ding[1], Li-Ping Feng[1], Qiong Shi[1]
1 Department of Computer Science and Technology, North University of China, Taiyuan, Shan'xi 030051, People's Republic of China

S1 File. Transversality condition.

Lemma 2.2 If condition (S1) is established, then the transversality condition

$$\left\{\mathrm{Re}\left(\frac{d\lambda}{d\tau}\right)\right\}_{\tau=\tau_{k_0}^{j}} > 0$$

is derived.

Proof: By taking the derivative on Eq. (10) with respect to τ, then we get

$$\begin{aligned}&\{2\lambda-[a_{11}+a_{22}+a_{23}e^{-\lambda\tau}-(d_1+d_2)k^2]\}\frac{d\lambda}{d\tau}+\{a_{23}\lambda+d_1a_{23}k^2\\&-(a_{11}a_{23}-a_{13}a_{21})\}\tau e^{-\lambda\tau}\frac{d\lambda}{d\tau}+[a_{23}\lambda+d_1a_{23}k^2-(a_{11}a_{23}-a_{13}a_{21})]\lambda e^{-\lambda\tau}=0,\end{aligned}$$

the above equality can be transformed into

$$\begin{aligned}\mathrm{Re}\left(\frac{d\lambda}{d\tau}\right)^{-1}_{\tau=\tau_{k_0}^{j}} =&\mathrm{Re}\left\{\frac{-2\lambda e^{\lambda\tau}+a_{11}e^{\lambda\tau}+a_{22}e^{\lambda\tau}+a_{23}-(d_1+d_2)k^2e^{\lambda\tau}}{[a_{23}\lambda+d_1a_{23}k^2-(a_{11}a_{23}-a_{13}a_{21})]\lambda}-\frac{\tau}{\lambda}\right\}_{\tau=\tau_{k_0}^{j}}\\
=&\mathrm{Re}\left\{\frac{-2\mathrm{i}w(\mathrm{cos}w\tau+\mathrm{isin}w\tau)+(a_{11}+a_{22})(\mathrm{cos}w\tau+\mathrm{isin}w\tau)}{\mathrm{i}w[a_{23}\mathrm{i}w+d_1a_{23}k^2-(a_{11}a_{23}-a_{13}a_{21})]}\right.\\
&\left.+\frac{a_{23}-(d_1+d_2)k^2(\mathrm{cos}w\tau+\mathrm{isin}w\tau)}{\mathrm{i}w[a_{23}\mathrm{i}w+d_1a_{23}k^2-(a_{11}a_{23}-a_{13}a_{21})]}\right\}_{\tau=\tau_{k_0}^{j}}\\
=&\frac{-2w_{k_0}^2[-w_{k_0}^2+d_1d_2k_0^4-d_2a_{11}k_0^2-d_1a_{22}k_0^2+a_{11}a_{22}]}{a_{23}^2w_{k_0}^4+[d_1a_{23}k_0^2-(a_{11}a_{23}-a_{13}a_{21})]^2w_{k_0}^2}\\
&+\frac{[(a_{11}+a_{22})-(d_1+d_2)k_0^2]^2w_{k_0}^2-a_{23}^2w_{k_0}^2}{a_{23}^2w_{k_0}^4+[d_1a_{23}k_0^2-(a_{11}a_{23}-a_{13}a_{21})]^2w_{k_0}^2}\\
=&\frac{[2w_{k_0}^2+(d_1^2+d_2^2)k_0^4-2d_1a_{11}k_0^2-2d_2a_{22}k_0^2+a_{11}^2+a_{22}^2-a_{23}^2)]w_{k_0}^2}{a_{23}^2w_{k_0}^4+[d_1a_{23}k_0^2-(a_{11}a_{23}-a_{13}a_{21})]^2w_{k_0}^2}\\
=&\frac{[2w_{k_0}^2+B_{k_0}]w_{k_0}^2}{a_{23}^2w_{k_0}^4+[d_1a_{23}k_0^2-(a_{11}a_{23}-a_{13}a_{21})]^2w_{k_0}^2}\\
=&\frac{w_{k_0}^2\sqrt{B_{k_0}^2-4C_{k_0}}}{a_{23}^2w_{k_0}^4+[d_1a_{23}k_0^2-(a_{11}a_{23}-a_{13}a_{21})]^2w_{k_0}^2}>0,\end{aligned}$$

thus, Lemma 2.2 is proved.
